# Environmental, socioeconomic, and health factors associated with gut microbiome species and strains in isolated Honduras villages

**DOI:** 10.1016/j.celrep.2024.114442

**Published:** 2024-07-04

**Authors:** Shivkumar Vishnempet Shridhar, Francesco Beghini, Marcus Alexander, Adarsh Singh, Rigoberto Matute Juárez, Ilana L. Brito, Nicholas A. Christakis

**Affiliations:** 1 Yale Institute for Network Science, Yale University, New Haven, CT, USA; 2 Department of Biomedical Engineering, Yale University, New Haven, CT, USA; 3 Meinig School of Biomedical Engineering, Cornell University, Ithaca, NY, USA; 4 Soluciones para Estudios de la Salud, Copán, Honduras; 5 Department of Statistics and Data Science, Yale University, New Haven, CT, USA; 6 Department of Medicine, Yale School of Medicine, New Haven, CT, USA

**Keywords:** gut microbiome species, gut microbiome strains, population-wide microbiome associations, non-western LMIC cohort, uncharacterized taxa, social networks, economic factors, polymorphic sites

## Abstract

Despite a growing interest in the gut microbiome of non-industrialized countries, data linking deeply sequenced microbiomes from such settings to diverse host phenotypes and situational factors remain uncommon. Using metagenomic data from a community-based cohort of 1,871 people from 19 isolated villages in the Mesoamerican highlands of western Honduras, we report associations between bacterial species and human phenotypes and factors. Among them, socioeconomic factors account for 51.44% of the total associations. Meta-analysis of species-level profiles across several datasets identified several species associated with body mass index, consistent with previous findings. Furthermore, the inclusion of strain-phylogenetic information modifies the overall relationship between the gut microbiome and the phenotypes, especially for some factors like household wealth (e.g., wealthier individuals harbor different strains of *Eubacterium rectale*). Our analysis suggests a role that gut microbiome surveillance can play in understanding broad features of individual and public health.

## Highlights

• Overall relationship between gut microbiome and host factors remains obscure
• We investigate factors associated with the gut microbiome in 1,871 rural Hondurans
• Socio-economic factors are the most pertinent relative to other host factors
• Different strains of various bacterial species are found in wealthier individuals

## Graphical abstract

**Figure:**
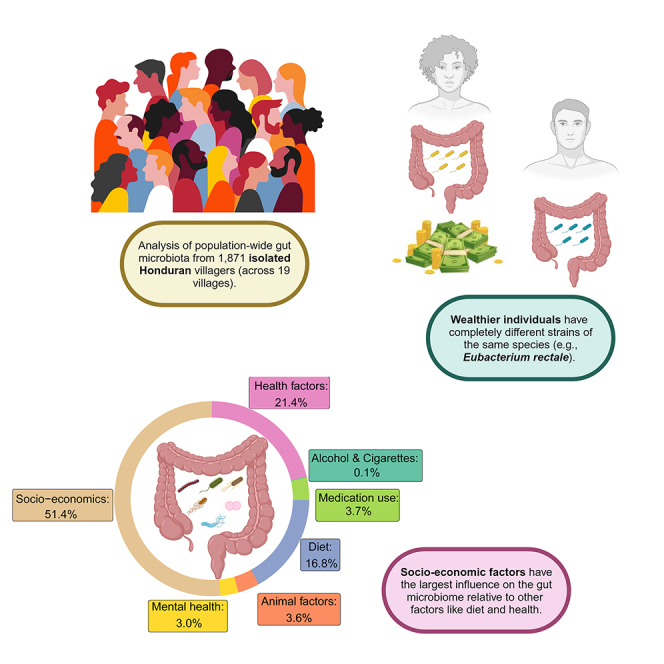

## Teaser

The complex relationship between the human gut microbiome and host factors remains obscure. Shridhar et al. implement a population-wide phenotype association analysis in rural Honduras and quantify microbes to the strain level. Socio-economic factors are the most influential in comparison to other well-studied factors like anthropometrics, chronic conditions, medication, and diet.

## Introduction

Thanks to long-run investments in gut microbiome research in industrialized countries, the role that the human microbiome plays in health-related phenotypes and its relationship to socioeconomic factors, and, reciprocally, how such phenotypes and factors might influence the microbiome, is becoming increasingly clear.^1^^,^^2^ For instance, an important prior study investigated such associations in a large cohort in the Netherlands, explicating these relationships.^1^

However, the majority of the human population lives outside of North America and Europe, and nearly half of the human population lives outside urban areas. Non-industrialized populations often experience problems with access to healthcare resources, have distinctive patterns of social interactions (e.g., low population density, fewer contacts with strangers), and have other distinctive exposures (e.g., animals and diet).^3^^,^^4^^,^^5^ Furthermore, prior studies of non-industrialized populations have documented the presence of rich uncharacterized taxa that are often absent in industrialized cohorts.^6^

Therefore, here, we investigate the relationships of both uncharacterized taxa and known species in a large sample drawn from an isolated setting in Honduras in order to describe the relationship of gut microbiome species and diverse attributes. We assessed 123 phenotypes and food, animal, and socioeconomic factors, and we compared selected outcomes with other Western and non-Western cohorts. Finally, we explored the role that strain-level information may play in these relationships—specifically, how influential factors like wealth or diet may drive strain-level variation in the gut microbiome.

## Results

### Isolated setting in western Honduras

**Figure 1:**
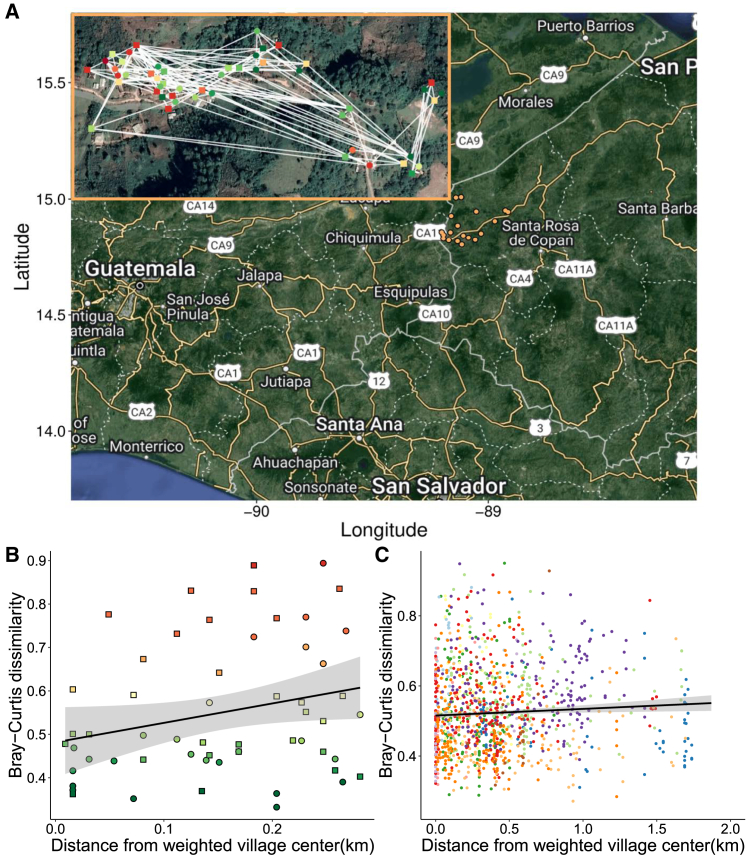
Geographic overview of the Honduras microbiome project (A) A satellite view of the Honduran villages (in orange) that constitute the microbiome dataset. In the inset, a zoomed-in satellite view of an illustrative village with each inhabitant (*n* = 57) colored with the respective Bray-Curtis dissimilarity value relative to the average microbiome composition of the rest of the village is shown and they are connected by white edges, which represent social interactions between individuals. Green nodes are indicative of higher similarity in microbiome composition to the rest of the village, whereas red nodes are more dissimilar. Square nodes indicate males, and circle nodes indicate females. (B) Scatterplot of Bray-Curtis dissimilarity (of the single village shown in A) and the distance of households from the population-weighted village centroid (see STAR Methods) shows a positive correlation (Pearson correlation coefficient ρ = 0.144, *p* = 0.05) between gut microbiome dissimilarity and distance from the village center across samples. Individual dots are colored according to the person’s dissimilarity from the village’s average microbiome. (C) Combined plot of all the Bray-Curtis dissimilarities and distances from village centroids for all villages’ inhabitants colored by village. The black regression line indicates a consistent trend (Pearson correlation coefficient ρ = 0.311, *p* = 2.2 × 10^−3^) of increasing microbiome dissimilarity with regard to the distance from the village centroid. The light gray areas indicate a 95% confidence interval.

The village communities in the western highlands of Honduras are geographically remote (Figure 1A), consisting of a large proportion of descendants of Mayan peoples who depend on subsistence agriculture and coffee cultivation. We collected population-level data in these small communities, including deep sequencing data and a comprehensive set of both individual and community-level characteristics regarding diverse socioeconomic, psychological, and health attributes. Our cohort consists of 1,871 people living in 19 villages, which are part of a larger cohort developed for a different original purpose.^7^^,^^8^

The adult population in our 19 villages ranges from 66 to 432 individuals. The average age of participants was 41 (SD = 17; range: 15–93), 63.7% were women, and 41.8% were married. Each of the 19 villages has its own intricately connected social networks with minimal inter-village contact, and they are separated not only by distance but also by elevation (Figure 1A).

Stool samples were collected for the 1,871 individuals and sequenced to characterize their microbiome composition. The average read depth is 82,082,675 reads (SD = 812,462.4) (Figure S1). Variations in microbiome composition can be appreciated even within the same village. For instance, we observed a pattern of decreasing similarity as individuals live farther away from the village center, at the geographic periphery of the village (Pearson correlation coefficient ρ = 0.311, *p* = 0.0022, Figures 1B and 1C). In contrast, villagers located at the network center of the social network within each village have a more similar microbiome to the rest of the village, unlike those at the social periphery (linear regression β = 3.66 × 10^−5^, *p* = 0.761; see STAR Methods for details and also the inset of Figure 1A).

### Species, phenotypes, and factors

Overall, we found 2,148 significant associations when looking at 639 microbial species and 123 factors (including physical and mental health, medication use, diet, animal exposure, and social and economic measurements; see Table S1). All comparisons involved appropriate statistical controls (see STAR Methods) and were corrected for multiple hypothesis testing using a false discovery rate procedure. Distinctly, we also found 988 associations with pathways (see Table S2).

The 123 factors are variously measured as continuous and discrete variables (Tables S3, S4, and S5), and, as expected, several of the variables were found to be correlated (for example, individuals with high hemoglobin A1c strongly correlated with reporting a diagnosis of diabetes, and the household wealth index correlated with owning a TV [Figure S2]). Similarly, the clustering of factors based on species effect sizes (obtained from the species-phenotype association models) showed that multiple factors within different categories have similar microbial signatures (Figure S3). Apart from individual phenotypes and factors, broader sub-categories of factors are correlated as well, like diet and economic factors, physiological variables and medication use, education and social factors, and so on. Food and animal factors also have a relative stronger correlation with socio-economic factors (Figure S2). Overall, this suggests that economic factors are intertwined with a broad array of factors (like health, food, animal, and other environmental variables), making them even more germane in the context of the gut microbiome in non-Western settings.

### Health phenotypes

**Figure 2:**
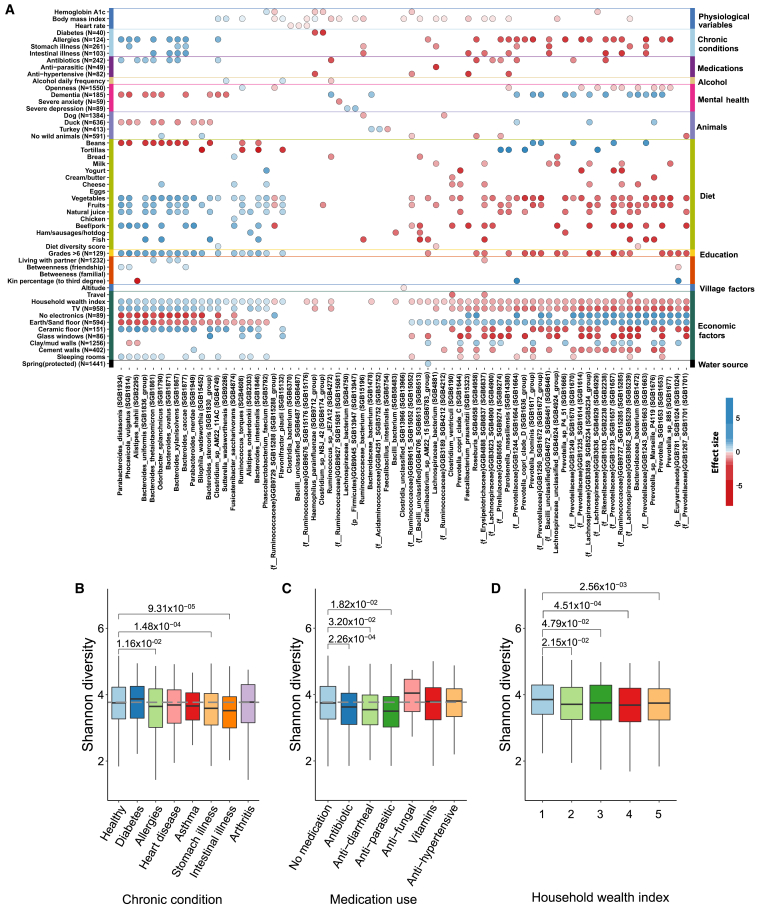
Microbiome association with factors (A) 81 species that best represent gut microbiome associations with 52 factors (chosen from health, food and animal, and socioeconomic categories; see Table S1 for a complete list of associations). The number of individuals manifesting the respective factor is shown in brackets. The presence of color shows significant associations for that phenotype-species pair (false discovery rate < 0.05); the intensity of the color corresponds to the strength of the effect size. Negative associations are indicated by red and positive by blue. Unknown species are indicated with “{}” specifying the taxonomic level at which the species is known. Listed factors without a sample size are reported for the whole sample. (B) Shannon diversity of healthy and chronically ill individuals highlights differences in overall microbiome diversity; healthy individuals (*n* = 1,407) are chosen as a reference (gray dashed line). (C) Shannon diversity is calculated between different medication use categories; non-medicated individuals (*n* = 1,246) are chosen as reference (gray dashed line). (D) Shannon diversity of villagers belonging to households classified by household wealth index ranging from 1 (least wealthy) to 5 (most wealthy). All comparisons were performed using the Wilcoxon rank-sum test and corrected for multiple hypothesis testing.

We found a total of 402 species to be significantly associated with at least one health phenotype (Tables S1 and S3). Among the 402 significant species, 302 of them belonged to the phylum Firmicutes, making it the most associated with health phenotypes. Among all the associated species, 34.58% were identified as unknown^9^ at several taxonomic levels. Species uSGB2239 from the Rikenellaceae family and *Parolsenella massiliensis* were the most frequently associated species, significantly associated with 5 health phenotypes; in particular, both were identified as negatively associated with body mass index (BMI), allergies, and intestinal illness (Figure 2A). uSGB2239 was also negatively associated with antibiotics and positively associated with dementia, and *Parolsenella massiliensis* was also associated with anti-hypertensive medication (negatively) and openness (negatively) (see STAR Methods). Microbial species from the Rikenellaceae family have been previously found to be associated with at least one mental health disorder (positively associated with obsessive-compulsive disorder)^10^ and enriched in type 2 diabetics in a Pakistani cohort.^11^ In another study, Rikenellaceae was found to be significantly associated with high blood sugar in Indian men.^12^ Coincidentally, we found that BMI was significantly associated with uSGB2239 of the Rikenellaceae family.

Furthermore, a total of 136 pathways were associated with at least one health phenotype, totaling 157 pathway associations. Among the 157 associations, physiological variables had 85 associations, followed by 24 associations in chronic illness phenotypes, 26 in medication, 2 in acute disease, and 19 in personality measures, alcohol, cigarettes, and mental health (Table S2).

We performed association analysis for a subset of individuals falling in unhealthy ranges of various health phenotypes (i.e., BMI <18 and BMI >25 to account for underweight and overweight individuals, respectively, or diastolic pressure >89 to account for hypertensive individuals) compared to healthy individuals (Figure S4; Table S6). A total of 73 species were associated with multiple unhealthy phenotypes, of which uSGB14313 of the Clostridia family was associated with 3 phenotypes in unhealthy ranges (hemoglobin A1c [5.7–6.4], BMI [25–30], and BMI [30–35]) (Figure S4A; Table S6).

Moving on from individual species, the diversity of an individual’s microbiome (measured with Shannon diversity) was computed, with an average alpha diversity of 3.7; incidentally, there was no significant difference in village-level alpha diversity (ANOVA *p* = 0.218). We evaluated whether the alpha diversity itself was associated with various health (and other) phenotypes. The majority of the villagers self-reported themselves as healthy (*n* = 1,407, 75.20%), and only 162 villagers (8.65%) reported having more than one disease. We observed that villagers with reported illnesses (except arthritis and diabetes) had lower diversity relative to healthy villagers (Figure 2B); in particular, villagers with reported stomach (Wilcoxon rank-sum test *p* = 1.48 × 10^−4^) and intestinal illnesses (Wilcoxon rank-sum test *p* = 9.31 × 10^−5^) had decreased diversity. Villagers who reported taking various medications also had lower diversity (Figure 2C); anti-parasitic drug users showed the lowest diversity (Wilcoxon rank-sum test *p* = 0.018), followed by anti-diarrheal users (Wilcoxon rank-sum test *p* = 0.032) and antibiotic users (Wilcoxon rank-sum test *p* = 2.26 × 10^−4^). We found no material associations of microbiome diversity with other categories of medications.

We also performed a contrast analysis by comparing the gut microbiome composition of these self-reported healthy individuals to individuals who reported at least one chronic condition by using differential abundance analysis, and we identified a total of 6 species that were differentially abundant between the two groups (Figure S4B; see STAR Methods). *Lachnospiraceae* bacterium (SGB4906) is the sole species found to be enriched in healthy individuals. On the other hand, uSGB1663 and uSGB27424 of the Prevotellaceae family, *Spirochaetia* bacterium, *Coprococcus*, and uSGB6369 of the Clostridia family were found to be enriched in diseased individuals.

Overall, all the health phenotypes put together contribute 5.7% of the total variance explained in microbial species composition (Figure S5; Table S7). Similarly, 11.6% of the variance in pathway composition is relevant to health phenotypes.

### Animal exposure and diet factors

We explored possible associations with animal exposure and diet.^1^^,^^13^^,^^14^^,^^15^ An unusual feature of our setting is that more than 90% of villagers reported having exposure to different types of animals, including wild animals, farm animals, and pets, affording possible zoonotic transmission. Overall, for all food and animal factors, 205 species were found to be significantly associated with at least one of the factors, resulting in 437 associations (Figure 2A; Table S4). Among all the associating bacterial species, 27% were unknown. Among the 205 significantly associated species, 122 of them belonged to Firmicutes, making this phylum the most commonly associated with specific animals or food categories. We found 10 pathways associated with exposure to animals as well (Table S2). Animal exposure contributed to 2.3% of the variation in species composition. We found no difference in overall Shannon diversity in individuals exposed to different animal categories (Figure S6).

Diet has been extensively studied and shown to have a substantial relationship with the gut microbiome.^15^^,^^16^^,^^17^ We assessed associations with microbial features and food frequency consumption and found 360 significant associations with diet (Figure 2A). *Bacteroides intestinalis* was the most associated species with food, associated with 8 different food types. In the past, *B. intestinalis* has been implicated in the context of dietary fiber as contributing to an increase of xylan utilization in the gut.^18^ Even though most of the individuals’ daily diet consists of tortillas and beans, we measured diet diversity using the diet diversity score (DDS)^19^ (see STAR Methods and Figure S7). We identified a total of 7 significant associations between the DDS and gut microbiome species (Figure 2A).

We also found 235 pathway associations with food factors (Table S2). Looking at significant associations between pathways and food factors, we found that the pathway L-histidine II degradation (PWY-5028) had a strong positive association with consumption of beef and pork. This pathway was also found to be enriched in humans consuming meat in a previous study,^20^ as dipeptides containing histidine are the major form of dipeptides in mammalian skeletal muscle.^21^ The role of biologically active peptides is highly correlated with consumption of beef (a protein-rich food) and its enrichment via gut microbiota.^22^

Overall, diet was responsible for 1.85% and 2.14% of the variance explained in our sample in species and pathways composition, respectively (Figure S5).

### Socioeconomic factors

Overall, we found 1,105 significant associations (51.44% of total associations) with socioeconomic factors. For all socioeconomic factors, 319 species were found to be significantly associated with at least one of the factors (Figure 2A; Table S1). Among all the 319 associated species, 28.8% of them were unknown, and 185 of them belong to Firmicutes, making it again the most associated phylum for socioeconomic factors. Moreover, uSGB5239 of the Lachnospiraceae family is the most-associated species, statistically significantly associated with 14 socioeconomic factors. We also found 586 associations with pathways, with one of them being associated with 9 socioeconomic factors (Table S2).

Socioeconomic factors are relevant to many exposures and personal habits. Higher monthly expenditures are correlated with a better diet and better household essentials such as a refrigerator or paved floor. We observed that most of the bacteria associated with higher monthly expenditures are the same as the ones associated with better diet quality.^23^^,^^24^

Although all the participants in our study are considered to be living in poverty, economic status still varied among them and was associated with possessions and diets potentially relevant to the microbiome; overall, the average household wealth index score (ranging from least wealthy [1] to most wealthy [5]) is 3.26 (SD = 1.33). In terms of measures of economic status, both monthly expenditure and travel were associated with the microbiome. Total wealth was also correlated with owning various items (such as a TV or a mobile phone), some of which (e.g., a refrigerator or a stove) might affect food consumption and others of which (such as having glass windows, cement walls, more sleeping rooms, an earthen floor, or a metal roof) might affect microbiome exposures via other routes (Figures 2A and S3). We observed similar patterns of association where a high wealth index was associated with the same bacterial species associated with owning expensive items (like glass windows), and vice versa. The variance explained by economic factors was 4.13% for species and 3.70% for pathways (Figure S5), indicating the relative importance of economic factors in explaining variation in the gut microbiome composition.

With respect to overall microbial diversity, the subjects from the least well-off households had a Shannon index that was higher than that of the subjects from the wealthier households (in the top 4 quintiles) (Figure 2D).

### Overall relationship between species and the phenotypes and factors

From the clustering of associations (Figure 2A) and the dendrogram (Figure S3), it can be observed that different factors can be linked together. This link can be visualized through the relationship between the gut microbiome and the factors. For example, species that are enriched in socioeconomic factors (such as TV ownership and household wealth index) show a similar pattern in vegetable, fruit, and meat consumption. Previous studies have found diet diversity to be correlated with food security and wealth in rural settings.^25^^,^^26^ Overall, health, food, animal, and socioeconomic factors are clustered together, which is also visibly demonstrated through the microbiome-phenotype lens. Multiple factors from different categories can also be tied together by a singles species. For instance, uSGB5239 (of the Lachnospiraceae family) is associated with 22 different phenotypes from the health category (BMI, stomach illness, dementia), the food and animal category (vegetables, fruits, natural juice, beef/pork, fish), and the socioeconomic category (grades >6, travel, household wealth index, TV, no electronics, earth/sand floor, ceramic floor, glass windows, clay/mud walls, cement walls, and sleeping rooms).^27^

### Comparison with other datasets and countries

**Figure 3:**
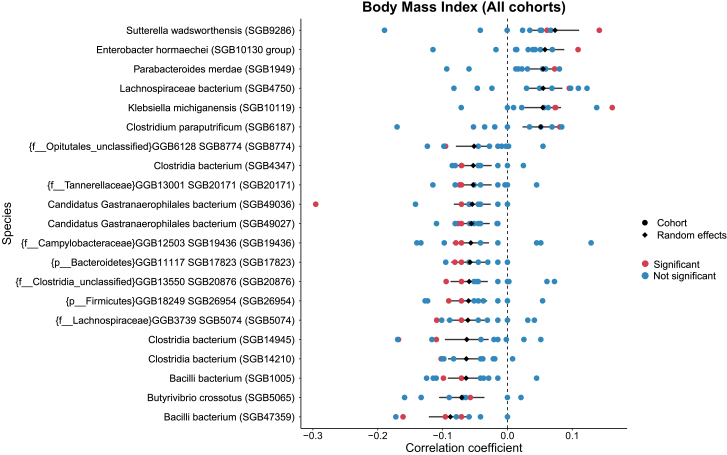
Meta-analysis of bacterial species associated with BMI across different cohorts Random-effect meta-analysis of BMI (body mass index) on 5,001 gut metagenomes species-level profiles across different Western and non-Western cohorts (points shown indicates correlation coefficients from each separate cohort). Species with statistically significant random effects estimates (*n* = 21) were included (see STAR Methods for more details and Table S11).

We compared microbial signature across datasets from other countries. Across the nine cohorts considered, we identified BMI to be the sole host phenotype shared across all of them.^6^^,^^28^^,^^29^^,^^30^^,^^31^^,^^32^^,^^33^^,^^34^^,^^35^ In our dataset, we also identified BMI to be one of the phenotypes with the most significant associations (*n* = 275). Therefore, a meta-analysis of BMI on 5,001 samples from the nine cohorts identified 21 significant species. *Sutterella wadsworthensis* (SGB9286) was found to be associated with a higher BMI in most of the datasets, while *Bacilli* bacterium (SGB47359) is the most negatively associated species (Figure 3). Coefficients from our cohort are statistically significant for all 21 species. Among other cohorts, one cohort in particular had the greatest number of significant coefficients (13 out of 21).^28^

Higher abundances of *Parabacteroides merdae* (SGB1949) have been found in obese mice and have also exhibited a protective effect against obesity-associated atherosclerosis.^36^ In human studies, *Butyrivibrio crossotus* (SGB5065) was found to be enriched in non-obese individuals,^37^ and *Sutterella wadsworthensis* (SGB9286; one of our strongest positively correlated species) was found to be 10 times more abundant in obese children-adolescents^38^—both of which were consistent with these meta-analysis findings.

Furthermore, we also compared our associations with the ones found by the Dutch Microbiome Project. We found 13 species-phenotype associations in common with the Dutch study, 8 of which were with BMI, and *Butyrivibrio crossotus*, *Roseburia inulinivorans*, *Faecalibacterium prausnitzii*, *Methanobrevibacter smithii*, *Eubacterium siraeum*, *Haemophilus parainfluenzae*, *Mitsuokella multacida*, and *Flavonifractor plautii* were found to be significantly associated in both datasets with BMI. Moreover, *Haemophilus parainfluenzae* was also significant for hemoglobin A1c in both datasets. *Ruminococcus torques* was significant in both datasets for antibiotic use. Finally, monthly income/expenditure had 3 significant species in common: *Alistipes shahii*, *Barnesiella intestinihominis*, and *Flavonifractor plautii*. The presence of very few significant species in common between the Honduras and Netherlands cohorts is largely due to differences in measurements between the two cohorts and the relatively low number of common species across the two cohorts (see Table S8 for a list of possible comparisons).

### Relevance of microbial strains

**Figure 4:**
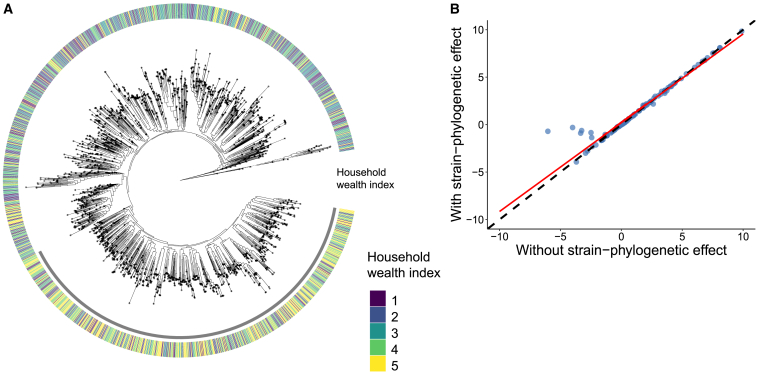
Microbial strain association with host factors (A) In this strain-level phylogeny of *Eubacterium rectale* (SGB4933 group) (as an illustrative microbe) in 1,610 individuals, leaves are annotated with the household wealth index (as an illustrative host factor). A cluster of individuals (annotated in gray) situated on a different strain of *Eubacterium rectale* (separated branch) are more likely to have higher wealth compared to rest of the individuals (Fisher’s exact test *p* = 0.0004998). (B) Comparison of significant effect sizes obtained from a linear mixed model with and without adding strain-level phylogeny information across significant species-phenotype relationships overall. Coefficients from the association models with and without phylogenetic information are positively correlated (Spearman correlation coefficient ρ = 0.989, *p* < 2.2 × 10^−16^), and the red line is the linear fit (β = 0.9351, intercept = 0.2016, *p* < 2.2 × 10^−16^), showing the relationship between the two models. The deviation of the red fitted line from the dashed line shows the important effect of adding the strain-level phylogeny in the species-phenotype association model (Table S9).

Finally, moving beyond species-specific associations with phenotypes and factors, we observed a meaningful variation between the genetic makeup of the same species across different individuals that is, in turn, associated with diverse factors (Figure 4A). For instance, individuals with a higher household wealth index are likely to have a different strain of *Eubacterium rectale* compared to less wealthy individuals in a set of 1,610 individuals (Fisher’s exact test *p* = 4.99 × 10^−4^) (Figure 4A).

Moreover, adding strain-phylogenetic information in the model alters the relationship between species and factors overall (Figure 4B) by inducing a small shift. Among all the effect sizes, 0.2% of them switch direction when adding the phylogenetic effect (Figure S8; Table S9).

**Figure 5:**
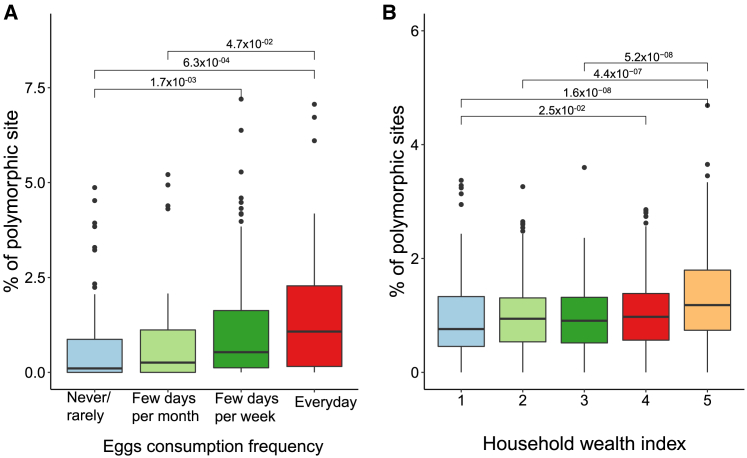
Relationship of variability in polymorphic sites with two host factors (A) Polymorphic site variability of uSGB4905 shows a gradual increase in the percentage of polymorphic sites in individuals consuming more eggs in their regular diet (*n* = 312). (B) Another demonstration of how variability in polymorphic sites changes with phenotype. Here, progressively wealthier individuals have a higher percentage of polymorphic sites in *Prevotella copriclade C* (SGB1644) (*n* = 1,458).

Looking deeper into the strain diversity in individuals, we evaulated the variation of the percentage of polymorphic sites across individuals and factors. As an illustration, we observed that wealthier individuals (β = 0.08345, *p* = 1.26 × 10^−7^) or those consuming a higher number of eggs (β = 0.626, *p* = 0.1146) had a higher percentage of polymorphic sites (Figures 5A and 5B; Table S10). This comports with findings in another study where percentages of polymorphic sites from just *Prevotella copri* strains were found to be different between recent South Asian Canadian immigrants and first-generation South Asian Canadians.^39^

## Discussion

Integrated, standardized, large, population-based cohorts to study the microbiome are uncommon, but such studies offer the prospect of identifying factors shaping the gut microbiome or being shaped by it. By extending our knowledge of the human gut microbiome to a novel population in a lower-and-middle-income (LMIC) setting, assessing previously uncharacterized taxa, having a very broad range of phenotypes and factors, and using strain-level genomic information, our goal is to advance understanding of the possible relationship of the gut microbiome with diverse human attributes.

We find that variation in the gut microbiome across individuals living in a traditional way in remote Honduran villages is partly explained by variations in diet, lifestyle, environment, and health factors. Overall, we found 2,148 unique associations between 639 bacterial species and 123 phenotypes/factors. Examining pairwise correlations between phenotypes and all gut microbial species, broader categories like food and animal factors were highly associated with socioeconomic factors, suggesting that wealth is an important underlying factor in this non-Western cohort. We also observe diet to be highly correlated with education and social factors. The associations between species and attributes included many uncharacterized species, which in many cases were shown to have a stronger effect than known species. Phenotype and factor associations were also identified after accounting for strain-level phylogenies, which often had a profound effect on the extent of the association between microbiome species and the attributes under consideration.

Still, despite measuring a large number and variety of factors, only 19.2% of the variation across individuals in microbiome composition was accounted for by these factors, in keeping with prior studies.^1^^,^^40^^,^^41^^,^^42^ This suggests that microbiome composition in individuals may be quite idiosyncratic or may depend on details of social interactions or unmeasured environmental exposures. Rare species may also help account for this variation. The current understanding of how individual and population-level microbiomes come to be shaped is thus still incomplete. Nevertheless, the factors we ascertained in Honduras did combine to account for 19.2% of the species variation (as noted) and 33.4% of the pathway variation; this may be compared to a study from the Netherlands where the measured phenotypes accounted for 13% and 16.2% of the variation, respectively,^1^ although different methodologies for taxonomic and functional characterization were used here, reflecting ongoing methodological advances. Shotgun metagenomic sequencing enabled us to further classify the functionality aspects of the gut microbiome, giving us a distinct advantage over 16S rRNA sequencing. Accurate profiling of the microbiome can be impacted by the choice of primers in the 16S method.^43^ Furthermore, in addition to these limitations to the 16S method, updated reference databases and tools enable us to profile a far greater number of species.^6^^,^^44^

It has already been established that the gut microbiome composition can be related to various health conditions in both humans and mice,^45^ and conditions like cancer, obesity, diabetes, anxiety, and depression can induce shifts in gut composition (as previously shown in many mostly Western populations).^1^^,^^45^^,^^46^^,^^47^^,^^48^^,^^49^^,^^50^^,^^51^ Alcohol intake and cigarette use have been linked to gut microbiome dysbiosis, as well as medications.^52^^,^^53^^,^^54^^,^^55^^,^^56^ In keeping with these prior studies, we confirm such findings in this rural LMIC cohort.^3^^,^^4^^,^^5^^,^^57^ Indeed, we found 606 associations between the microbiome and health-related phenotypes and factors. Chronic illnesses and medication use were the most strongly associated. Among chronic illnesses, intestinal illnesses show the greatest differences. We uncovered 273 total associations between gut microbiome species and physiological measurement ranges that may be linked to underlying chronic conditions such as obesity, diabetes, and hypertension. Moreover, we found 62 associations with mental health phenotypes alone, a relatively understudied area.

Comparisons with other non-Western cohorts can highlight some of the differences and similarities between Honduras and such cohorts as well. Comparing BMIs across 5 other non-Western cohorts (from India, Cameroon [2], Peru, and Madagascar) and 4 western cohorts (Great Britain/USA, USA [2], and China) using a meta-analysis approach, we found 21 species significantly associated with BMI in all cohorts, among which the Honduran correlation coefficients were consistently significant and close to the unbiased estimate, reflecting stable yet significant associations because of the sample size.

Looking at the overall microbial composition among healthy and chronically ill subjects, the Shannon diversity was generally lower in chronically ill people, especially those with allergies and gastrointestinal illnesses. Moreover, comparing healthy individuals to those who are chronically ill, we found 6 taxa to be differentially enriched in one of the groups. *Lachnospiraceae* bacterium is the only significant species differentially enriched in healthy individuals. On the other hand, 2 unknown and 3 known species, uSGB1663 and uSGB27424 of the Prevotellaceae family, *Spirochaetia* bacterium, *Coprococcus*, and uSGB6369 of the Clostridia family, were differentially enriched in chronically diseased individuals. Among medication users, those taking anti-parasitic medication had the largest drop in overall diversity.

Another factor that greatly influences the gut microbiome is diet. Our sample population exhibits a consistent diet, with beans and tortillas consumed by most people daily. Still, we found 360 associations with food categories. Moreover, just as a previously studied Dutch cohort found that pets had notable associations with the microbiome,^1^ we likewise found 77 associations with a (broader) range of animal exposures.

Social and economic factors had 1,105 associations, with the bulk of the strong associations coming from unknown species. The gut microbiome samples had 947 unique associations with economic factors alone, making it the second highest associated category of variables we examined, after health. Wealth differences in individuals can also manifest in the form of more diverse strains in some species being present in wealthier individuals. Prior research in Honduras has highlighted the crucial importance of socioeconomic status in addressing health in such communities,^58^ and the microbiome varies in important ways along this axis, even in this poor setting.

Social interaction is an integral part of Honduran villagers’ life. In total, 123 unique associations with various social network factors were found. Studies investigating social interactions between mice have shown the evolutionary advantage of having behaviors that enhance social interaction that consequently facilitates microbiome transmission.^45^^,^^59^^,^^60^ In wild mice, social associations are predictive of microbiome composition, and the microbiome is correlated across mice interaction networks.^61^ In humans, strain-level similarities have been shown in familial and partner networks within and outside households.^62^^,^^63^^,^^64^ Whether these interactions translate into exposures that directly contribute to health is an important area for further work.

Finally, our samples were collected from individuals spread across 19 villages separated in space and elevation, and the overall gut microbiome samples were observed to vary with the relative spatial position within the villages; the dissimilarity score with a village-averaged microbiome increased as subjects lived further away from the village center. Relatedly, we found 3 significant associations with elevation.

Uncharacterized taxa play a vital role in all these associations, as in prior LMIC cohorts.^6^ Despite the number of unknown species in the Honduran cohort being about a third of total species, their relative strength of associations was observed to be higher in all the phenotype/factor categories. Distinctly, strain-level information is also relevant to the microbiome-phenotype relationship and should, optimally, be accounted for.

### Limitations of the study

To fully understand the effects of host factors like diet and environment, surveying the exact quantity of various food groups (in addition to the food frequency questionnaire) could improve the accuracy of our associations of diet with gut microbiome. As for environmental factors, investigating the exact sources of water and food shared between individuals could help more accurately answer the impact of a shared environment on the gut microbiome in comparison to other host factors. And, of course, our findings arise from the analysis of data from a single region of the world.

### Conclusions

These findings advance understanding the interplay between various phenotypes and host factors on the one hand, and the gut microbiome on the other. By expanding our knowledge of the human microbiome to a novel non-Western cohort, it is possible to further our understanding of the role of the gut microbiome in chronic illness and, at the same time, open up opportunities to use such findings to develop inexpensive biomarkers to aid diagnostics in rural settings.^65^^,^^66^^,^^67^ To the extent that a healthy microbiome is driven by modifiable social and environmental factors (such as diet, smoking, living arrangements, lifestyle, and so on), understanding which factors to target or what possible microbiome-modifying interventions to implement could help advance individual and collective health in diverse settings.

## STAR★Methods

### Key resources table

**Table:** 

REAGENT or RESOURCE	SOURCE	IDENTIFIER
**Biological samples**		

Human stool samples from Honduran cohort	This Paper	N/A

**Critical commercial assays**		

TissueLyzer	Qiagen, Hilden, Germany	N/A
Chemagic Stool gDNA extraction kit	Perkin Elmer, Massachusetts, USA	N/A
KAPA Hyper Library Preparation	KAPA Biosystems, Massachusetts, USA	N/A
Illumina NovaSeq 6000	Illumina, California, USA	N/A

**Deposited data**		

Raw human gut metagenomic sequencing data	This paper	NCBI-SRA, accession number: PRJNA999635

**Software and algorithms**		

Prinseq lite	Cantu et al.^68^	https://github.com/uwb-linux/prinseq
BMTagger	BMTagger^69^	https://ftp.ncbi.nlm.nih.gov/pub/agarwala/bmtagger/
Trimmomatic	Bolger^70^	https://github.com/usadellab/Trimmomati
Trellis	Lungeanu et al.^71^	https://trellis.yale.edu/
MetaPhlAn (version 4.0.0)	Blanco-Míguez et al.^9^	https://github.com/biobakery/MetaPhlAn/
StrainPhlAn (version 4.0.0)	Blanco-Míguez et al.^9^	https://github.com/biobakery/MetaPhlAn/
VEGAN (version 2.3–5)	Dixon^72^	https://CRAN.R-project.org/package=vegan
HUMAnN (version 3.0.0)	Beghini et al.^73^	https://github.com/biobakery/humann
ggmap	Kahle and Wickham^74^	https://github.com/dkahle/ggmap
lmerTest (version 3.1.0)	Kuznetsova et al.^75^	https://CRAN.R-project.org/package=lmerTest
Meta (version 4.9–9)	Balduzzi et al.^76^	https://CRAN.R-project.org/package=meta
Evolvability (version 2.0.0)	Hansen et al.^77^	https://CRAN.R-project.org/package=evolvability
MaAsLin2 (version 1.0.0)	Mallick et al.^78^	https://github.com/biobakery/Maaslin2
Phytools (version 1.9–23)	Revell^79^	https://github.com/liamrevell/phytools
Available code for this study	This paper	https://github.com/human-nature-lab/Phenotype-paper. (Zenodo: https://doi.org/10.5281/zenodo.11476406)

### Resource availability

#### Lead contact

Further information and requests should be directed to Dr. Nicholas A. Christakis (nicholas.christakis@yale.edu).

#### Materials availability

This study did not generate new unique reagents.

#### Data and code availability

• Metagenomic sequences for the study participants are deposited in NBCI SRA and available under accession number PRJNA999635.
• The code for replicating the analysis is available at https://github.com/human-nature-lab/Phenotype-paper. (Zenodo https://doi.org/10.5281/zenodo.11476406).
• Any additional information required to reanalyze the data reported in this work paper is available from the lead contact upon request.

### Experiment model and study participation details

#### Sample collection, library preparation, and sequencing

Participants were instructed on how to self-collect the fecal samples using a training module and promptly returned samples to a local team which then stored them in liquid nitrogen at the collection site and then moved them to a −80 C° freezer in Copan Ruinas, Honduras. Samples were then shipped on dry ice to the United States and stored in −80 C° freezers.

Stool material was homogenized using TissueLyzer from Qiagen and the resulting lysate was prepared for extraction with the Chemagic Stool gDNA extraction kit (PerkinElmer) and extracted on the Chemagic 360 Instrument (PerkinElmer) following the manufacturer’s protocol. Sequencing libraries were prepared using the KAPA Hyper Library Preparation kit (KAPA Biosystems). Shotgun metagenomic sequencing was carried out on Illumina NovaSeq 6000. Samples not reaching the desired sequencing depth of 50Gbp were re-sequenced on a separate run.

Raw metagenomic reads were deduplicated using prinseq lite (version 0.20.2^68^) with default parameters. The resulting reads were screened for human contamination (hg19) with BMTagger^69^ and then quality filtered with trimmomatic^70^ (version 0.36, parameters “ILLUMINACLIP:nextera_truseq_adapters.fasta:2:30:10:8:true SLIDINGWINDOW:4:15 LEADING:3 TRAILING:3 MINLEN:50”).

This resulted in a total of 1,871 samples with an average read depth is 82,082,675 (SD = 812,462.4) (Figure S1). The adult population in our 19 villages ranges from 66 to 432 individuals. The average age of participants was 41 (SD = 17; range: 15–93); 63.7% were women; and 41.8% were married. The average household wealth index score (ranging from least wealthy (1) to most wealthy (5)) is 3.26 (standard deviation 1.33), measured from various household items.

#### Local involvement in the research

In keeping with proper standards for such research, we worked closely with the local population of Copan, sought feedback and approval from officials at the Ministry of Health (MOH) of Honduras, and endeavored to provide practical benefits to the local community. Here, we briefly summarize this history and outline some of our principles and actions in this regard.^8^

When we began designing this cohort project in 2013 (for the whole cohort of 176 villages and 24,702 people in the parent RCT), the Bill and Melinda Gates Foundation (BMGF) introduced us to the Inter-American Development Bank (IDB), which has been supporting and doing work throughout Latin America, and IDB in turn introduced us to the Honduras MOH. Because of this pathway to getting the project launched, we worked with local and regional public health organizations and with local leaders. From the outset when the original underlying cohort for this study was impaneled, we sought extensive local involvement, beginning with a needs assessment where local village residents told us about topics of concern to them in a series of meetings in villages throughout the Copan region.

We periodically briefed both the communities and the MOH about our findings. We also provided other material benefits to the local community. When we tested people for parasites as part of our study, we gave them the results of their tests and arranged for them to be treated. When we tested people for vision, we provided corrective glasses. We solicited ideas from the local community about what infrastructure improvements we could make, and we repaired many local playgrounds and clinics as a result. We arranged for an American company to provide free portable handheld ultrasound devices to the local health clinics, which was much appreciated by local providers. In terms of capacity building, we hired and trained over 100 local people and built capacity in the region.

Throughout our work in Honduras, and given the extent of local involvement at the regional and MOH levels, we endeavored to act with integrity, curiosity, and respect in all relationships.

Finally, we note that this research would not have been prohibited in the USA. This work is not likely to result in stigmatization, incrimination, or discrimination for the participants, and we have carefully safeguarded all data from threats to the privacy or security of our participants, which has constrained the individual-level data released here.

### Method details

#### Taxonomic profiling and diversity analysis

Quantification of organisms’ relative abundance was performed using MetaPhlAn 4,^9^ which internally mapped the metagenomes against a database of ∼5.1M marker genes describing more than 27k∼ species-level genome bins (SGB).

We identified a total of 2,508 species in our dataset. Among the 2,508 species, 639 species were used for association analysis after filtering for minimum relative abundance values (10^−2^), and a minimum of 10% prevalence in the population (*n* = 187).

We performed strain-level profiling for these species with StrainPhlAn 4^9^(parameters: “-phylophlan_mode accurate”)

Microbiome species richness was estimated using the Shannon entropy index and the total number of observed species (i.e., those with relative abundance simply greater than zero). Multidimensional scaling analysis (cmdscale R function) was performed on the Bray-Curtis dissimilarity index (vegdist function from the vegan R package^72^) calculated on the relative abundances obtained by MetaPhlAn4.

Functional potential analysis was performed using HUMAnN 3.0.^73^ Gene family profiles were normalized using relative abundances and collapsed into MetaCyc pathways.

To understand the amount of variance explained by various factors, we performed a PERMANOVA analysis (adonis function from the vegan package^72^) using the “bray” method; the diversity matrix was calculated on both species-level relative abundances and MetaCYC pathway relative abundances as input, including the 123 phenotypes variables into the model. All the comparisons were run with 999 permutations.

#### Factor characterization

We measured a broad range of phenotypes and factors using standard measures.^7^ Description and statistics on all factors can be found in Table S2. Summary of associations between pathways and phenotypes, Table S3. Metadata summary of health phenotypes, Table S4. Metadata summary of food and animal phenotypes. Physiological measurements were deemed within normal limits in accordance with CDC^81^ and NBME^82^ guidelines (Figure S3).

We used self-reported information to discern whether people were healthy or were diagnosed with various conditions. General anxiety disorder is derived from a set of 7 questions from a self-reported survey-based questionnaire though our TRELLIS software,^71^ which assigns a score of 0 to “Not at all”, 1 to “Several days”, 2 to “More than half the days”, and 3 to “Nearly every day”. The scores are added up (maximum of 21) and partitioned as: Minimal or none (≤5), Mild (6–10), Moderate (11–15), and Severe (≥16).^83^ The PHQ9 (Patient Health Questionnaire) score measuring depression was computed similarly, with the levels being: Minimal or none (≤5), Mild (6–10), Moderate (11–15), Moderately severe (16–19), and Severe (≥20).^84^ Personality traits like Openness or Nervousness were also based on self-reported questions, where the participants were asked to rate themselves between strongly disagree to strongly agree for each of the personality questions.

The Frequency of intake of various food items was self-reported, ranging from: “Never/rarely” to “Every day”. These frequencies were used as input in the diet-microbiome association model. The diet diversity score (DDS)^19^ was calculated by classifying individual food types into one of the following categories: cereals, roots/tubers, vegetables, fruits, meat/poultry/offal, eggs, fish/seafood, pulses/legumes/nuts, milk and dairy products, oils/fats, or sugar/honey. If any of these food items were consumed daily, the respective categories would get 1 for that individual. The sum across these categories would define the DDS score of this individual. The maximum possible DDS score would be 11 and the minimum would be 0.

Numerical values were reported for alcohol frequency and cigarette frequency. The daily alcohol intake ranged from “1 or 2” to “10 or more” drinks. Cigarette usage was reported as a “Yes” or “No”.

The household wealth index is computed using Multiple Correspondence Analysis (MCA) based on all the household items. The index ranged from 1, indicating low wealth, to 5, indicating high wealth.

We explored associations with several social network features, including the degree, transitivity, and betweenness centrality of each individual. To uncouple the effects of kin and non-kin social connections, we investigated microbiome associations in familial networks, friendship networks, and combined networks. In the combined network, we computed the amount of kin in a person’s first 3 degrees of social connections (i.e., among a person’s friends, friends of friends, and friends of friends of friends) to assess the relative effect of having kin close to a person within the social network. In addition to kin and non-kin relationships, we also explored the microbiome’s association with cohabiting partners.

#### Population-weighted village centroid

We collected the GPS coordinates (latitude and longitudes) of all the building in the village. Since multiple individuals can reside in a building, the population-weighted centroid was chosen as the reference center of the village, which was then used to compute every individual’s distance from this village center. Satellite plots were created using “ggmap” package in R.^74^

### Quantification and statistical analysis

#### Model for microbiome-factor regression

For the association model with species-level microbiome and the factors, a linear mixed-effects model was used to explore the relationship of the variability in the factors and the variability in the microbiome. The linear mixed-effect models were created using the lmerTest R package (v 3.1).^75^

For every species and phenotype pair, we computed the following model:

Speciesabundance∼Factorofinterest+Age+Sex+BMI+BatchEffect+BristolStoolScale+DNAconcentration+Samplingdate+(1|VillageID)

Species-level relative abundances were transformed using the CLR (Centered-Log Ratio) and used as input.

Since basic demographic attributes (age, sex), technical factors (DNA concentration, sequencing batch, sampling date), and BMI and Bristol stool scale accounted for most of the species and pathway variation, we used those variables as primary controls in our association models (Figures S5 and S9).

Furthermore, all associations were corrected for both microbiome species and factor using multiple hypothesis testing (Benjamini-Hochberg correction) and all significant associations are corrected for a FDR (False Discovery Rate) < 0.05.

#### Meta-analysis of BMI across non-Western cohorts

We screened publicly available datasets using the curatedMetagenomicData package (v3.6.2)^80^ to look for cohorts from similar populations and sharing the most number of available metadata. We identified a total of 5 non-western studies having in common BMI^6^^,^^29^^,^^30^^,^^34^^,^^35^ along with 4 western cohorts^28^^,^^31^^,^^32^^,^^33^ amounting to 5,001 samples. Data was downloaded from NCBI SRA using the accessions available through ‘curatedMetagenomicData’ and processed using the same pipeline described before.

We then performed a meta-analysis on BMI values using species-level relative abundances using. Age, gender, and lifestyle category were used as controls. We discretized age by binning the value into three levels: child-adolescent (<18), adult (18–60), and senior (>60).

Also, a random effect meta-analysis was performed using species-level relative abundances normalized with CLR using the meta package (v 4.9–9,^76^). After using linear model to obtain correlation coefficients, the metacor function (from meta package) was used to Random effects using Paule-Mandel estimator method. *p*-values obtained were adjusted using FDR (Benjamini-Hochberg corrected). In total, 21 species were found significant after corrections. The full results are available in Table S11.

#### Strain-factor analysis and phylogenetic signal

For strain-level analysis, we used the Almer function from the “evolvability” R package (v 2.0.0).^77^ Almer incorporates phylogenetic trees in mixed linear models as a correlated random effects structure.

Speciesabundance∼Phenotypeofinterest+Age+Sex+BMI+Batcheffect+BristolStoolScale+DNAconcentration+Samplingdate+(1|VillageID)+(1|phyl)

where, “phyl” is the variance-covariance matrix calculated from the species’ phylogenetic tree. To evaluate the strain-phylogenetic effect, we compared beta coefficients from this model and the same model without the random effect on the variance-covariance matrix.

The phylogenetic signal was estimated using the “phylosig” function in “phytools” R package (v 1.9–23)^79^ using the ‘lambda’ method. Overall, among the 78,597 species-phenotype pairs (639 species and 123 phenotypes), 52,864 pairs were chosen after filtering for phylogenetic signal. The phylogenetic signal was estimated for the phylogenetic tree of each species versus the phenotype of interest.

#### Polymorphic sites analysis

For polymorphic sites, files suffixed with “.polymorphic” in StrainPhlAn 4 output were used after discarding 0’s in the “percentage of polymorphic sites” column so as to discard subjects without the species of interest. Wilcoxon rank-sum tests were performed across categories within phenotypes to check for significant changes in polymorphic sites. In addition, linear regression was also performed to investigate the relationship between polymorphic site percentage and individual host phenotypes (see Table S10).

#### Differential abundance analysis

We used MaAsLin2 (v 1.0.0)^78^ to determine the association between species and disease status (healthy or unhealthy) of individuals and to estimate the effect sizes and *p*-values. Statistically significant species were retained. Species-level relative abundances were normalized using CLR and used as input for MaAsLin2. Age, sex, BMI, DNA concentration, sampling date, and Bristol stool scale were used as fixed-effect controls and village as a random effect control. All the resulting *p*-values obtained from the MaAsLin2 models were corrected for multiple hypothesis testing using FDR.

## Supplemental information

Document S1. Figures S1–S9

Table S1. Summary of associations between species and phenotypes Multivariate mixed effect linear regression between CLR transformed species (from MetaPhlAn 4) abundances and host-phenotypes of interest. (see STAR Methods). Age, sex, BMI, Bristol stool scale, sampling date, batch effect, and DNA concentration are the covariates/fixed effects, and village is a random effect in the model. Effect sizes, p-values, and FDR-adjusted p-values for each pair of species-phenotypes are provided. In total 78,597 separate regressions were performed (639 species times 123 phenotypes).

Table S2. Summary of associations between pathways and phenotypes Multivariate mixed effect linear regression between CLR transformed pathway abundances (from HUMAnN 3) and host-phenotypes of interest. Age, sex, BMI, Bristol stool scale, sampling date, batch effect, and DNA concentration are the covariates/fixed effects and village is a random effect in the model. Effect sizes, p-values, and FDR-adjusted p-values for each pair of species-phenotypes are provided. In total 60,270 separate regressions were performed (490 pathways times 123 phenotypes).

Table S3. Metadata summary of health phenotypes Summary description of health phenotypes stratified by sex and subcategories for all 1,871 individuals. (reported as percentages or mean (SD)). Subcategories include Physiological variables, Overall health, Acute conditions, Chronic conditions, Medication use, Personality types, Cognitive variables, Unfavorable habits, Anxiety, and Depression. Personality types have category types (agree strongly, agree, neither agree nor disagree, disagree, disagree strongly) (see STAR Methods). Anxiety and Depression have mild, moderate, and severe ranges in their respective scales (GAD7 and PHQ9, see STAR Methods).

Table S4. Metadata summary of food and animal phenotypes Summary description of food and animal phenotypes stratified by sex and subcategories for all 1,871 individuals. (reported as percentages or mean (SD)). Animal exposures are reported by percentage for all 3 sub-categories (Pets, Farm animals, and Wild animals). Individual food items are reported from FFQ (Food Frequency Questionnaire, see STAR Methods).

Table S5. Metadata summary of socioeconomic phenotypes Summary description of socio-economic phenotypes stratified by sex and subcategories for all 1,871 individuals. (reported as percentages or mean (SD)). Social phenotypes include sub-categories like Partners, Education, and social-network factors (kin & non-kin). Education has three levels: (Primary (1–3), Middle (4–6), and Secondary (>6). Environmental factors include risky behavior and village factors. Economic factors include Income, Household essentials, and Water sources. The household wealth index is constructed based on household essentials.

Table S6. Summary of associations between species and unhealthy phenotypes Multivariate mixed effect linear regression between CLR transformed species abundances (from MetaPhlAn 4) and unhealthy ranges of health phenotypes of interest. All unhealthy ranges were considered as separate levels in each regression, with healthy being the reference level. Age, sex, BMI, Bristol stool scale, sampling date, batch effect, and DNA concentration are the covariates/fixed effects, and Village is a random effect in the model. Effect sizes, p-values, and FDR-adjusted p-values for each pair of species-phenotypes are provided. In total 78,597 separate regressions were performed (639 species times 123 phenotypes). Unhealthy ranges in Hemoglobin A1c can be categorized as: Diabetic (>7, 6.5–7) and Pre-diabetic (5.7–6.4). Unhealthy ranges in Blood pressure can be categorized as Elevated (Systolic (>119) & Diastolic (≤79), High blood pressure/Hypertension stage 1 (Systolic (>119) & Diastolic (>79)), and Hypertension Stage 2 (Diastolic (>89) or Systolic (>129)). Unhealthy ranges in Body Mass Index (BMI) can be categorized as Underweight (<18), Over-weight (25–30), Obese (30–35), and Morbidly Obese (>35). Unhealthy range in Heart rate is > 100. Unhealthy ranges in Oxygen saturation include (<97). Unhealthy ranges in total Hemoglobin can be categorized as Elevated (male>18 or female>16) and Low (male<13 or female<12).

Table S7. Variance explained in the gut microbiome Breakdown of the species and pathways variation explained by each sub-category belonging to health, food and animal, and socio-economic host phenotypes. Analysis was performed using PERMANOVA (999 permutations) (see STAR Methods).

Table S8. Comparison between Honduran and Dutch datasets Comparison of effect sizes, p-values, and FDR-adjusted p-values for each pair of species-phenotypes in the Honduran and the Dutch (LifeLines) datasets were closely examined for common associations. In total 13 significant associations were common between the two datasets. The disparity in the datasets is due to: (i) Different numbers of species (due to different versions of MetaPhlAn used in profiling species abundances) which led to 70 species being common to both datasets. (ii) Differences in measurements of host phenotypes across all categories which led to 16 common phenotypes (see Figure S9).

Table S9. Summary of associations between species and phenotypes (along with strains) Multivariate mixed effect linear regression coefficients between CLR transformed species abundances (from MetaPhlAn 4) and host-phenotypes of interest in samples having strain-phylogenetic information (from StrainPhlAn 4). (see STAR Methods). Strain phylogenies were constructed by StrainPhlAn for each species, resulting in a tree comprising of individuals based on the genetic makeup of the species in their respective guts. In addition to species-phenotype associations being performed (in samples present in the phylogenetic trees), the Almer package was used to add strain-phylogenetic information in the species-association model. Overall, each species-phenotype association was contrasted with and without considering strain phylogeny. Age, Sex, BMI, Bristol stool scale, sampling date, batch effect, and DNA concentration are the covariates/fixed effects, and village is a random effect in the model. Effect sizes, p-values, and FDR-adjusted p-values for each pair of species-phenotypes are provided. In total, 62,361 separate regressions were performed (507 species times 123 phenotypes). To avoid noise, StrainPhlAn trees with more than 100 samples were considered, resulting in 507 species.

Table S10. Summary of associations between polymorphic sites and phenotypes Multivariate mixed effect linear regression between polymorphic sites (from StrainPhlAn 4) and host-phenotypes of interest. (see STAR Methods). Polymorphic variation across samples was considered as a dependent variable in the regression model against host-phenotypes. Species-phenotype association was contrasted with and without strains. Effect sizes, p-values, and FDR-adjusted p-values for each pair of species-phenotypes are provided. In total 78,597 separate regressions were performed (639 species times 123 phenotypes).

Table S11. Summary statistics from meta-analysis of BMI for every species Species-level meta-analysis coefficients. A meta-analysis on BMI was performed on 5 non-western and 4 western cohorts apart from this Honduran cohort (5,001 samples in total) accounting for 3,444 species. We also report meta-analysis statistics (standard error, confidence interval, Z-score, I^2^, tau and p-value) as well as dataset-level correlation coefficients.

Document S2. Article plus supplemental information
