# Supplementary material for: Environmental, socioeconomic, and health factors associated with gut microbiome species and strains in isolated Honduras villages
Source: Cell Rep. 2024 Jul 4;43(7):114442. doi: 10.1016/j.celrep.2024.114442 (PMC11290354; doi:10.1016/j.celrep.2024.114442)
Supplement: Document S1. Figures S1–S9 [file mmc1.pdf]

Cell Reports, Volume *43*

Supplemental information

Environmental, socioeconomic, and health factors associated with gut microbiome species and strains in isolated Honduras villages

Shivkumar Vishnempet Shridhar, Francesco Beghini, Marcus Alexander, Adarsh Singh, Rigoberto Matute Juárez, Ilana L. Brito, and Nicholas A. Christakis

Figures List:

1. **Honduran Gut microbiome description**
2. **Phenotype-phenotype correlation.**
3. **Phenotype-microbiome association clustering.**
4. **Relationship between health and microbiome.**
5. **Variance explained.**
6. **Alpha diversity of individuals exposed to animals.**
7. **Diet diversity score.**
8. **Comparison of species and strain models.**
9. **Principal Coordinates Analysis (PCoA) of the full Honduran cohort.**

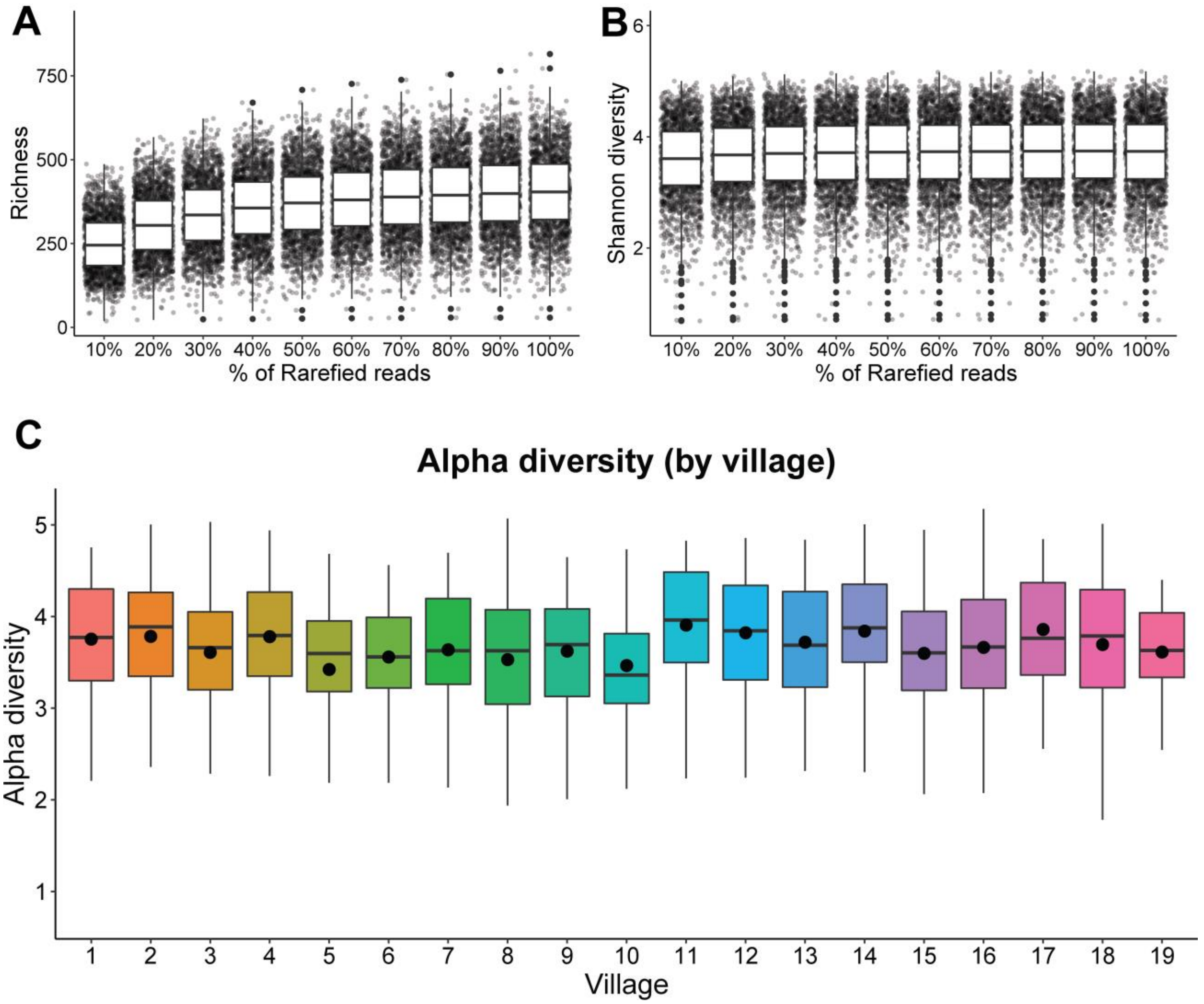

Figure S1 Honduran Gut microbiome description (Related to STAR methods). (A) Collector's curve of species richness (number of species per sample) across varying percentages of rarefied reads for all 1,871 Honduran samples (in light dots and box plot) show an increasing trend followed by a plateau at 60% of the all the rarefied reads. The average species richness is around 380 (plateau). (B) Collector's curve of alpha-diversity vs percentage of read-depth across all 1,871 Honduran samples show a flat trend. (C) The overall average of Alpha diversity across cohort is 3.7 and there is no village statistically different in distribution of alpha diversity compared to any other village (Wilcoxon rank-sum test). Black dots indicate mean alpha diversity for each village.

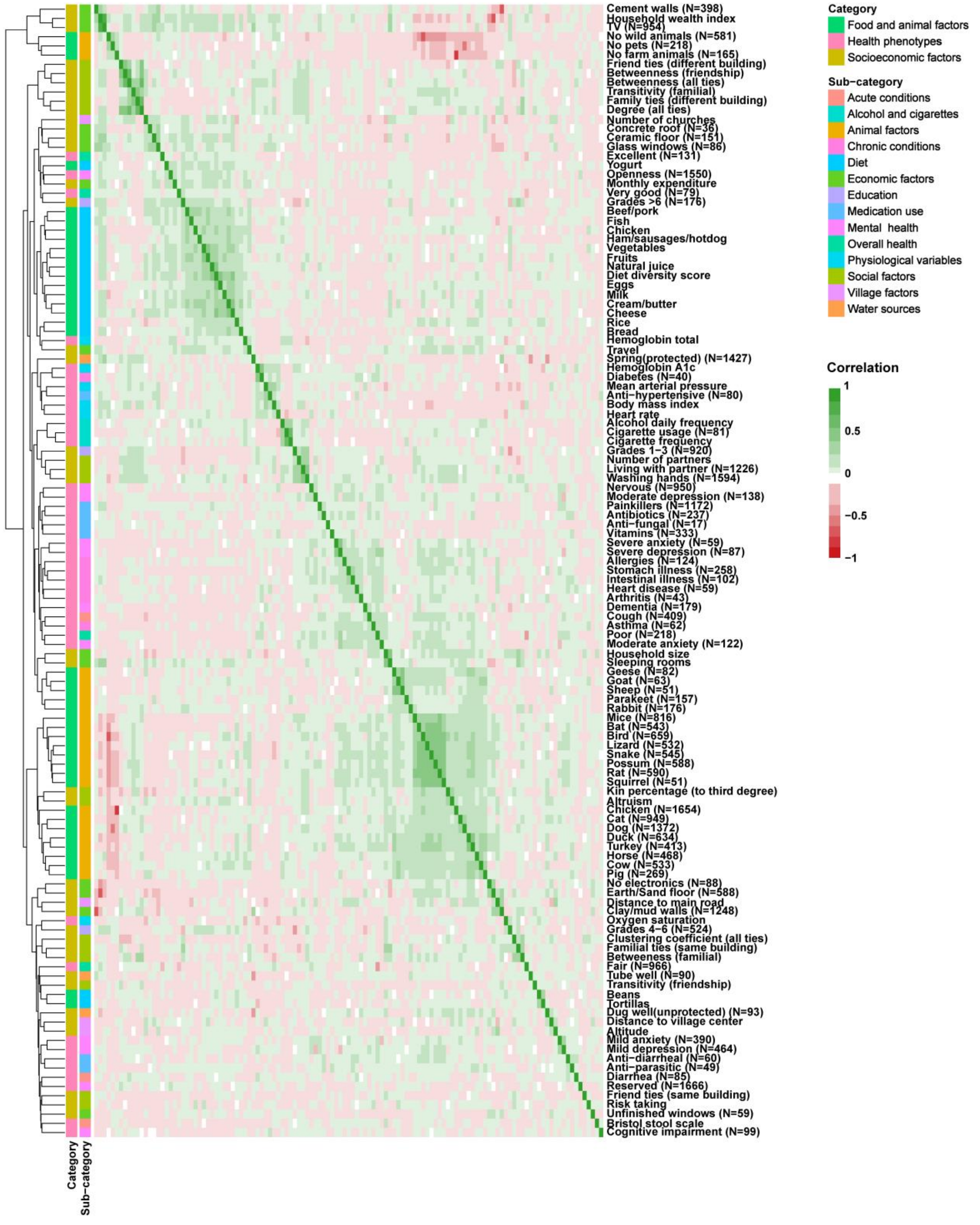

Figure S2 Phenotype-phenotype correlation (Related to Figure 2). (A) A matrix showing raw correlations between the phenotypes from every category (health, food and animals, socioeconomic factors). Column names are the same as the row names indicated on the right side of the matrix. Color ranges from positive (green) to negative (red) correlations. The correlations are also clustered according to the hierarchical clustering and annotated according to broader category or sub-category of phenotypes and factors (see **Supplementary table 1**).

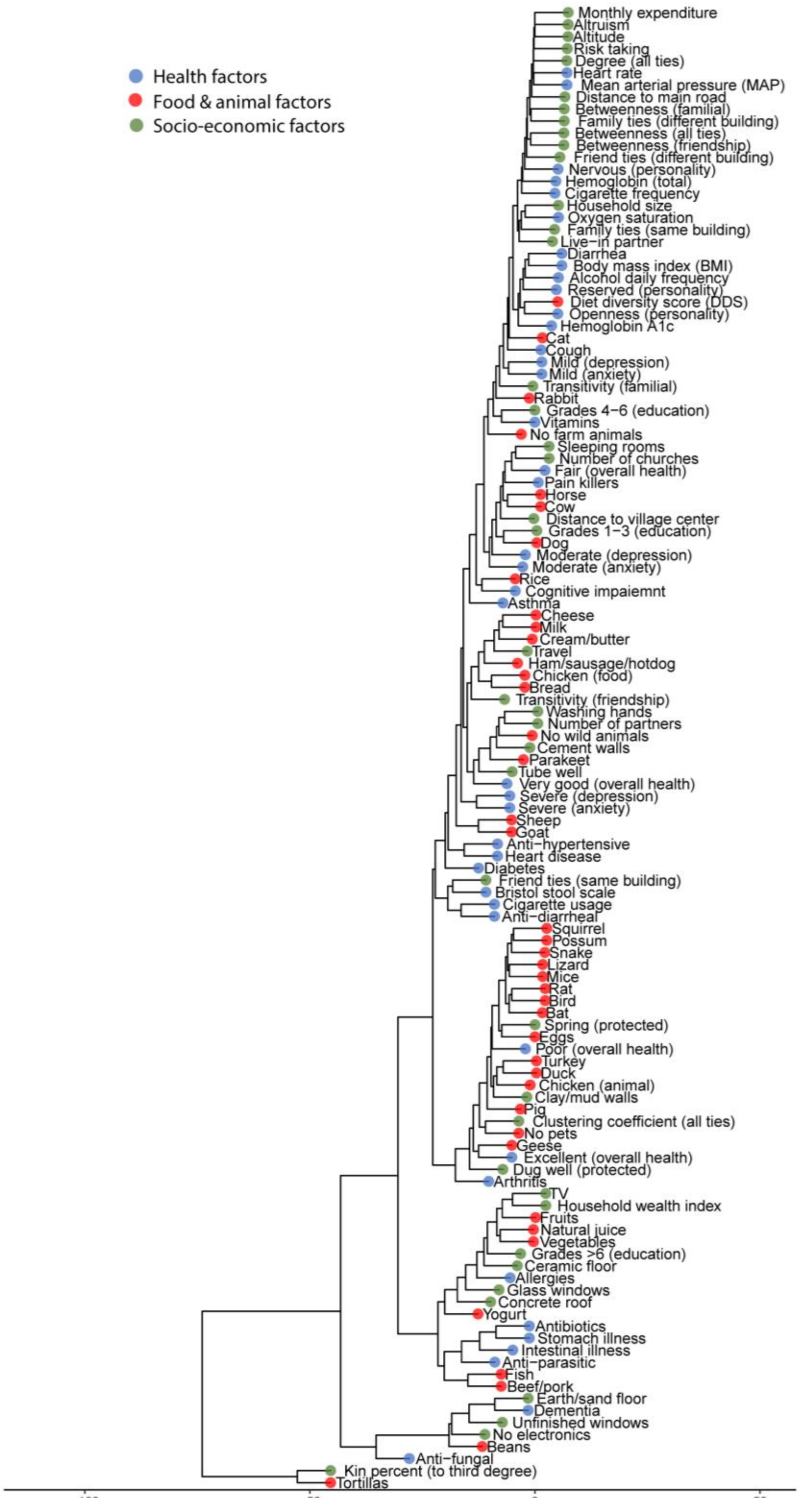

Figure S3 Phenotype-microbiome association clustering (Related to Figure 2). Effect sizes from associations of all 123 phenotypes with 639 species are hierarchically clustered with respect to phenotypes. This phenotype tree is another representation of how similarly behaving a pair of phenotypes are with respect to how they associate with the gut microbiome overall.

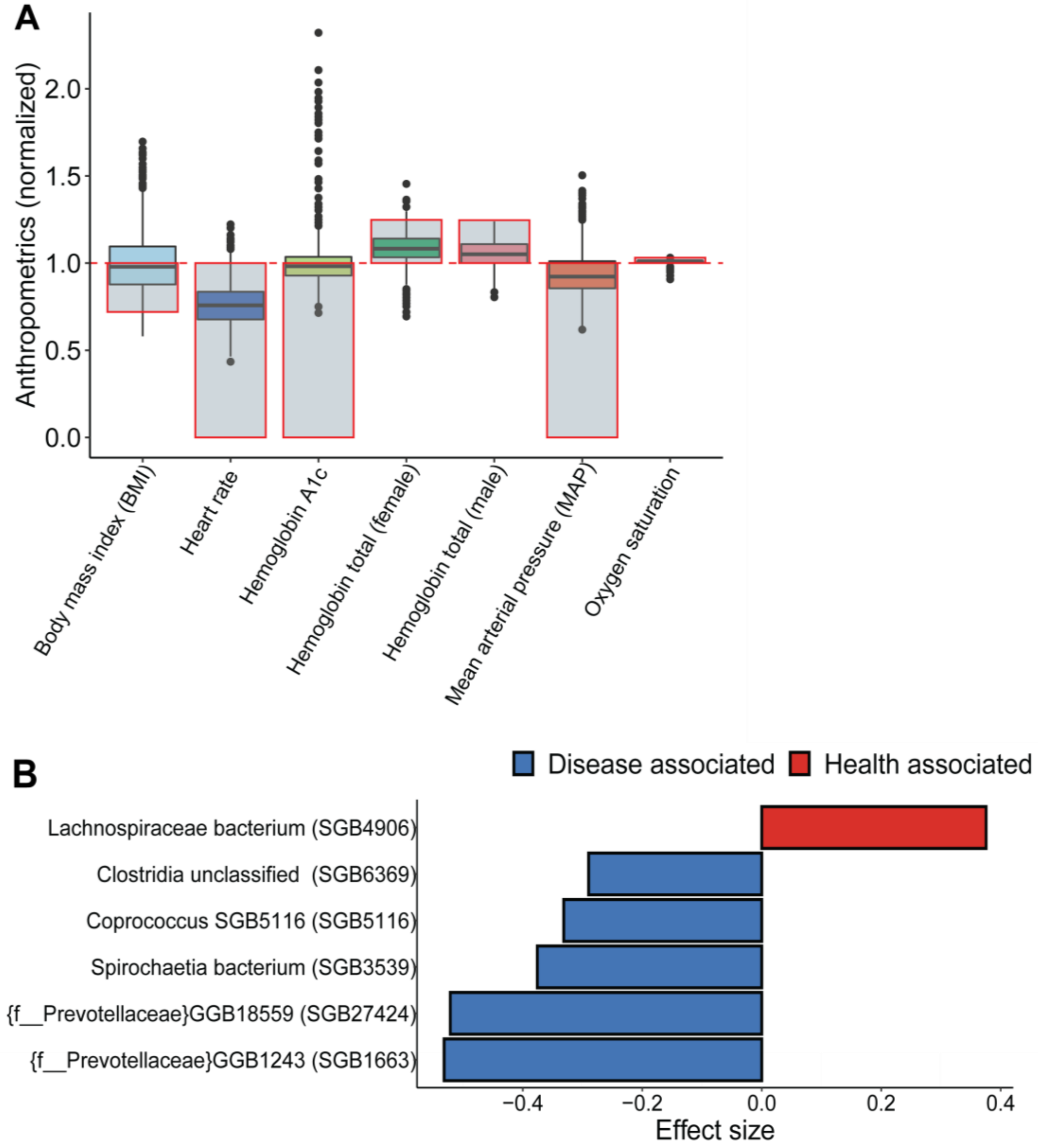

Figure S4 Relationship between health and microbiome (Related to STAR methods). (A) Graphical visualization of physiological measurements (anthropometrics) of all N=1,871 villagers, with the grey box indicating normal values of each respective physiological measurement. The red box indicates the bounding limit of healthy ranges. (B) In the entire cohort, there were 468 chronically diseased individuals (who had at least one chronic condition). Differential abundance in healthy vs chronically diseased individuals using MaAsLin2 (see **Methods**) shows six significant species (after FDR correction of p-values). One of them (*Lacnospiraceae bacterium*) was differentially abundant in healthy individuals. On the other hand, five species (uSGB1663 and uSGB27424 from the *Prevotellaceae* family, *Spirochaetia bacterium*, *Coprococcus*, and uSGB6369 from the *Clostridia* family), two of which are unknown, were differentially abundant in chronically diseased individuals.

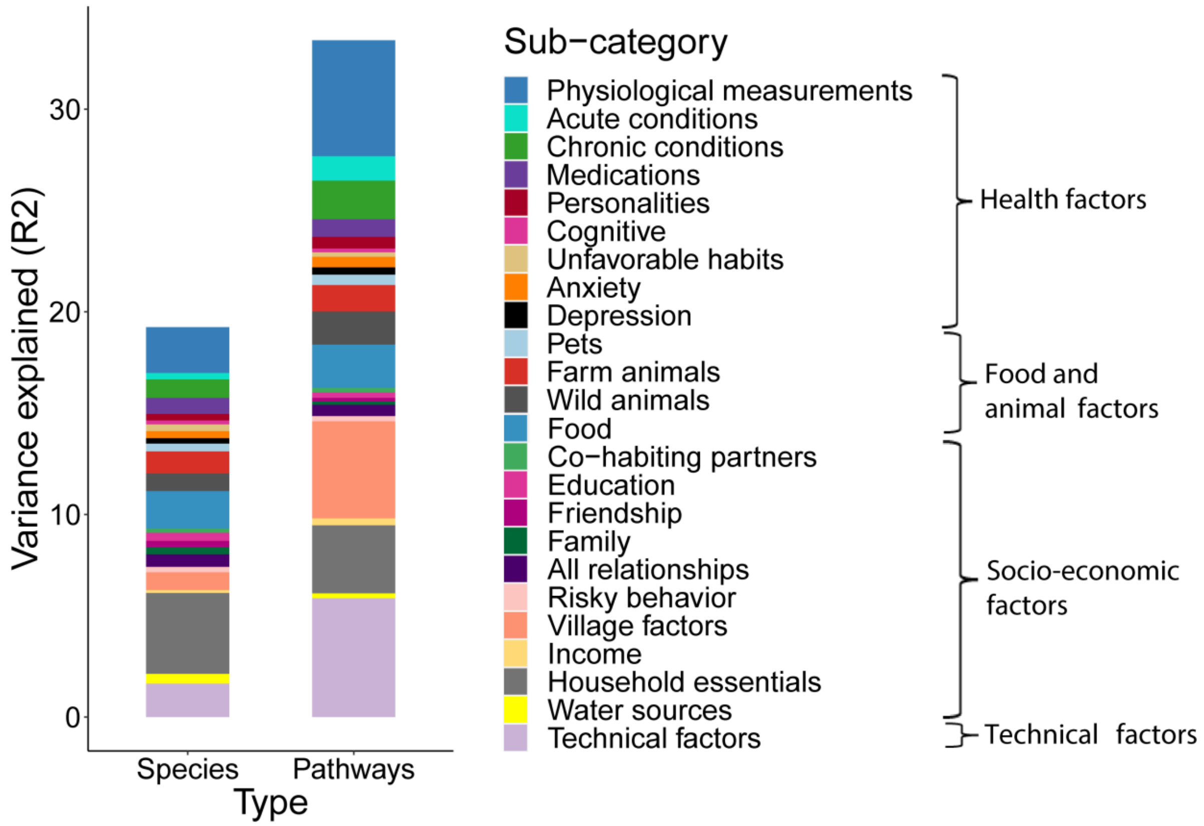

Figure S5 Variance explained (Related to Figure 2). PERMANOVA analysis (999 permutations, p-value<0.001) computed on all phenotypes shows the variance explained in species and pathway compositions with a breakdown of sub-categories of all phenotypes (health, food and animal, socioeconomic factors). Overall, all the phenotypes together explain 19.2% and 33.4% of the variance explained in species and pathways, respectively. "Technical factors" here include age, sex, DNA concentration, sequencing batch, and sampling date. (See **Supplementary Table 7** for complete breakdown of variance explained in each sub-categories)

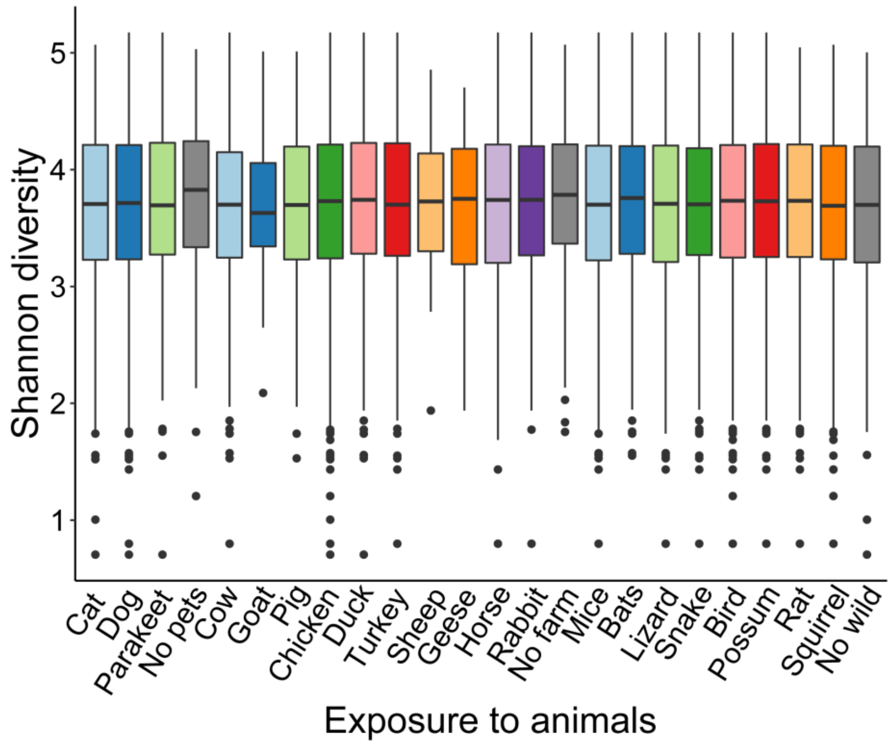

Figure S6: Alpha diversity of individuals exposed to animals (Related to Figure 2). Shannon diversity distribution among villagers exposed to pets, farm animals, and wild animals shows no significant differences between groups.

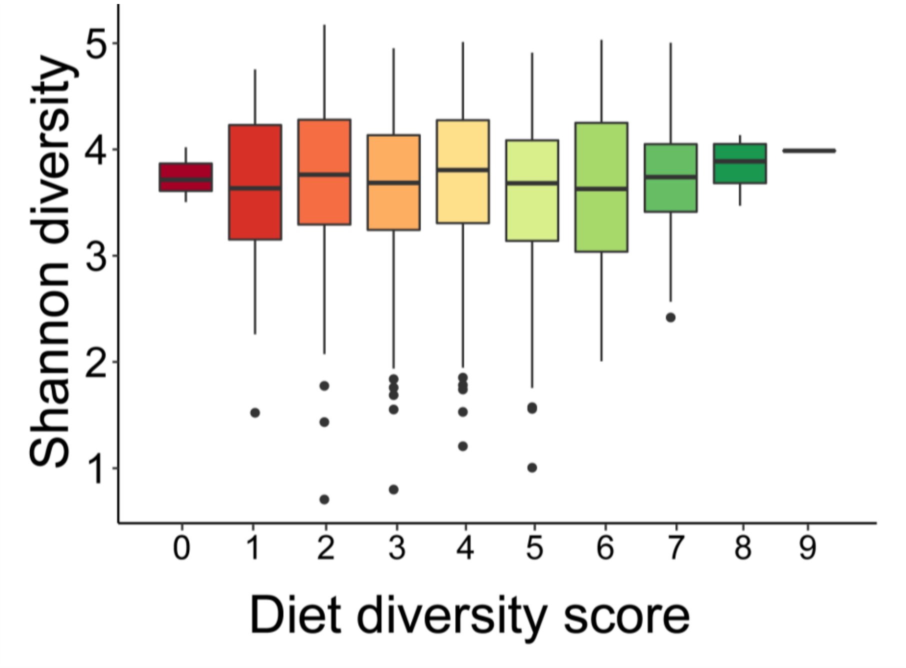

Figure S7: Diet diversity score (Related to Figure 2 and STAR methods). Boxplot showing the Shannon diversities of individuals with varying diet diversity scores (see **STAR Methods** for calculation of DDS scores).

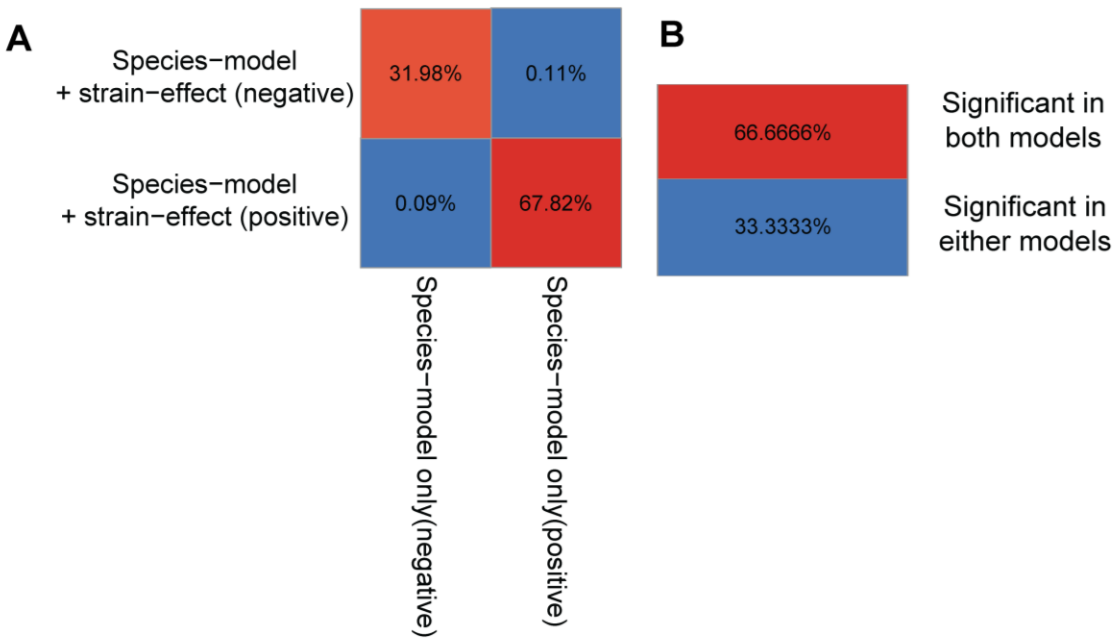

Figure S8: Comparison of species and strain models (Related to Figure 4). (A) Side-by-side comparison of the direction of associations in both models (with and without strain-phylogenies). Each quadrant indicates positive or negative associations in either model. (B) Figure showing the presence of significant associations in both models compared to their presence in either of the models.

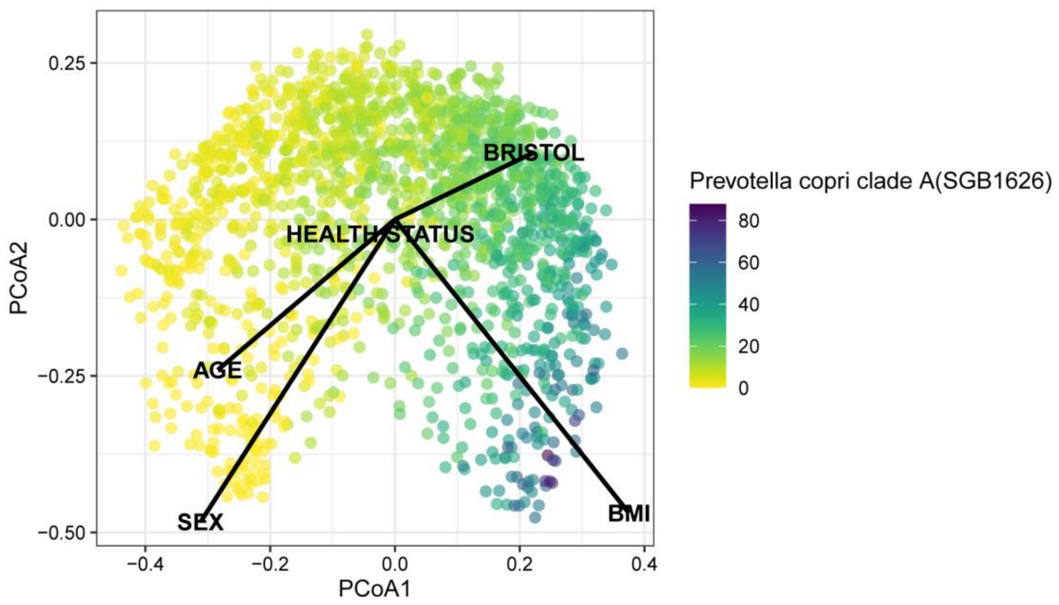

Figure S9: Principal Coordinates Analysis (PCoA) (Related to STAR methods). PCoA plot of the overall gut microbiome computed across 1,871 samples using the species-level relative abundances (legend) generated by MetaPhlAn4. Health status, age, sex, body mass index (BMI), and Bristol stool scale are shown as arrows along with the direction of influence. Samples are colored with the relative abundances of *Prevotella copri* (clade A).
