# Supplementary material for: Environmental, socioeconomic, and health factors associated with gut microbiome species and strains in isolated Honduras villages
Source: Cell Rep. 2024 Jul 4;43(7):114442. doi: 10.1016/j.celrep.2024.114442 (PMC11290354; doi:10.1016/j.celrep.2024.114442)
Supplement: Document S2. Article plus supplemental information [file mmc13.pdf]

Cell Reports

Article

Environmental, socioeconomic, and health factors associated with gut microbiome species and strains in isolated Honduras villages

Graphical abstract

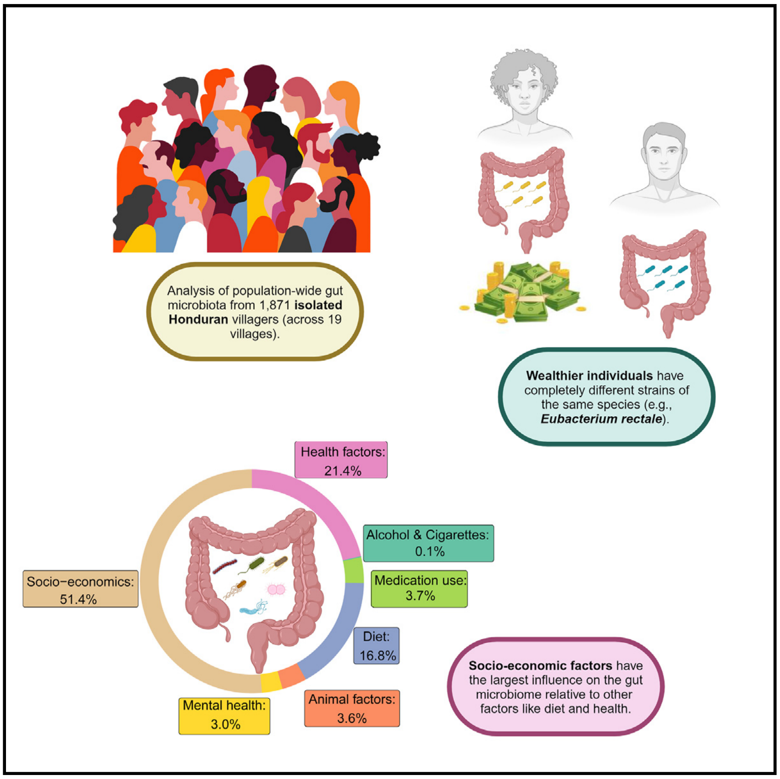

Highlights

- Overall relationship between gut microbiome and host factors remains obscure
- We investigate factors associated with the gut microbiome in 1,871 rural Hondurans
- Socio-economic factors are the most pertinent relative to other host factors
- Different strains of various bacterial species are found in wealthier individuals

Authors

Shivkumar Vishnempet Shridhar, Francesco Beghini, Marcus Alexander, Adarsh Singh, Rigoberto Matute Juárez, Ilana L. Brito, Nicholas A. Christakis

Correspondence

ibrito@cornell.edu (I.L.B.),
nicholas.christakis@yale.edu (N.A.C.)

In brief

The complex relationship between the human gut microbiome and host factors remains obscure. Shridhar et al. implement a population-wide phenotype association analysis in rural Honduras and quantify microbes to the strain level. Socio-economic factors are the most influential in comparison to other well-studied factors like anthropometrics, chronic conditions, medication, and diet.

Shridhar et al., 2024, Cell Reports *43*, 114442
July 23, 2024 © 2024 The Authors. Published by Elsevier Inc.
https://doi.org/10.1016/j.celrep.2024.114442

Cell Reports

CellPress
OPEN ACCESS

Article

Environmental, socioeconomic, and health factors associated with gut microbiome species and strains in isolated Honduras villages

Shivkumar Vishnempet Shridhar,[1,2,7] Francesco Beghini,[1,7] Marcus Alexander,[1] Adarsh Singh,[3] Rigoberto Matute Juárez,[4] Ilana L. Brito,[3,8,*] and Nicholas A. Christakis[1,2,5,6,8,9,*]
[1]Yale Institute for Network Science, Yale University, New Haven, CT, USA
[2]Department of Biomedical Engineering, Yale University, New Haven, CT, USA
[3]Meinig School of Biomedical Engineering, Cornell University, Ithaca, NY, USA
[4]Soluciones para Estudios de la Salud, Copán, Honduras
[5]Department of Statistics and Data Science, Yale University, New Haven, CT, USA
[6]Department of Medicine, Yale School of Medicine, New Haven, CT, USA
[7]These authors contributed equally
[8]Senior author
[9]Lead contact
*Correspondence: ibrito@cornell.edu (I.L.B.), nicholas.christakis@yale.edu (N.A.C.)
https://doi.org/10.1016/j.celrep.2024.114442

SUMMARY

Despite a growing interest in the gut microbiome of non-industrialized countries, data linking deeply sequenced microbiomes from such settings to diverse host phenotypes and situational factors remain uncommon. Using metagenomic data from a community-based cohort of 1,871 people from 19 isolated villages in the Mesoamerican highlands of western Honduras, we report associations between bacterial species and human phenotypes and factors. Among them, socioeconomic factors account for 51.44% of the total associations. Meta-analysis of species-level profiles across several datasets identified several species associated with body mass index, consistent with previous findings. Furthermore, the inclusion of strain-phylogenetic information modifies the overall relationship between the gut microbiome and the phenotypes, especially for some factors like household wealth (e.g., wealthier individuals harbor different strains of *Eubacterium rectale*). Our analysis suggests a role that gut microbiome surveillance can play in understanding broad features of individual and public health.

INTRODUCTION

Thanks to long-run investments in gut microbiome research in industrialized countries, the role that the human microbiome plays in health-related phenotypes and its relationship to socioeconomic factors, and, reciprocally, how such phenotypes and factors might influence the microbiome, is becoming increasingly clear.[1,2] For instance, an important prior study investigated such associations in a large cohort in the Netherlands, explicating these relationships.[1]

However, the majority of the human population lives outside of North America and Europe, and nearly half of the human population lives outside urban areas. Non-industrialized populations often experience problems with access to healthcare resources, have distinctive patterns of social interactions (e.g., low population density, fewer contacts with strangers), and have other distinctive exposures (e.g., animals and diet).[3–5] Furthermore, prior studies of non-industrialized populations have documented the presence of rich uncharacterized taxa that are often absent in industrialized cohorts.[6]

Therefore, here, we investigate the relationships of both uncharacterized taxa and known species in a large sample drawn from an isolated setting in Honduras in order to describe the relationship of gut microbiome species and diverse attributes. We assessed 123 phenotypes and food, animal, and socioeconomic factors, and we compared selected outcomes with other Western and non-Western cohorts. Finally, we explored the role that strain-level information may play in these relationships—specifically, how influential factors like wealth or diet may drive strain-level variation in the gut microbiome.

RESULTS

Isolated setting in western Honduras

The village communities in the western highlands of Honduras are geographically remote (Figure 1A), consisting of a large proportion of descendants of Mayan peoples who depend on subsistence agriculture and coffee cultivation. We collected population-level data in these small communities, including deep sequencing data and a comprehensive set of both individual and community-level characteristics regarding diverse

© 2024 The Authors. Published by Elsevier Inc.
This is an open access article under the CC BY license (http://creativecommons.org/licenses/by/4.0/).

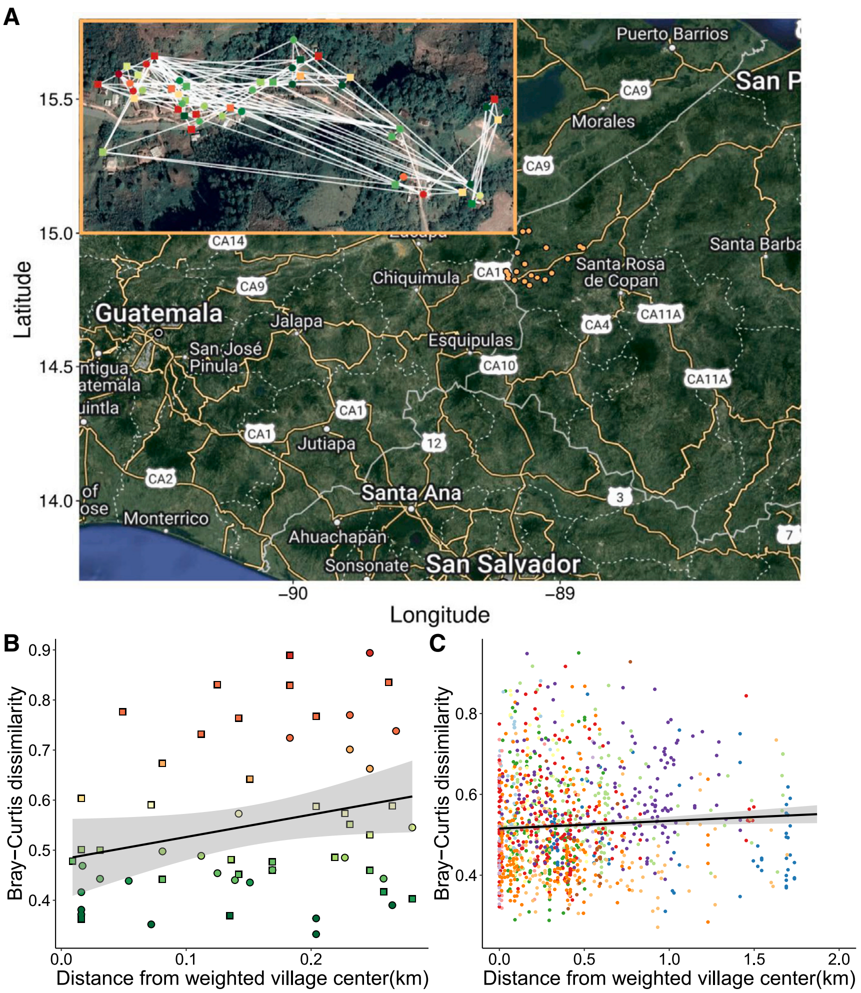

Figure 1. Geographic overview of the Honduras microbiome project

(A) A satellite view of the Honduran villages (in orange) that constitute the microbiome dataset. In the inset, a zoomed-in satellite view of an illustrative village with each inhabitant (n = 57) colored with the respective Bray-Curtis dissimilarity value relative to the average microbiome composition of the rest of the village is shown and they are connected by white edges, which represent social interactions between individuals. Green nodes are indicative of higher similarity in microbiome composition to the rest of the village, whereas red nodes are more dissimilar. Square nodes indicate males, and circle nodes indicate females.

(B) Scatterplot of Bray-Curtis dissimilarity (of the single village shown in A) and the distance of households from the population-weighted village centroid (see STAR Methods) shows a positive correlation (Pearson correlation coefficient ρ = 0.144, p = 0.05) between gut microbiome dissimilarity and distance from the village center across samples. Individual dots are colored according to the person's dissimilarity from the village's average microbiome.

(C) Combined plot of all the Bray-Curtis dissimilarities and distances from village centroids for all villages' inhabitants colored by village. The black regression line indicates a consistent trend (Pearson correlation coefficient ρ = 0.311, $p = 2.2 \times 10^{-3}$) of increasing microbiome dissimilarity with regard to the distance from the village centroid. The light gray areas indicate a 95% confidence interval.

socioeconomic, psychological, and health attributes. Our cohort consists of 1,871 people living in 19 villages, which are part of a larger cohort developed for a different original purpose.[7,8]

The adult population in our 19 villages ranges from 66 to 432 individuals. The average age of participants was 41 (SD = 17; range: 15–93), 63.7% were women, and 41.8% were married. Each of the 19 villages has its own intricately connected social networks with minimal inter-village contact, and they are separated not only by distance but also by elevation (Figure 1A).

Stool samples were collected for the 1,871 individuals and sequenced to characterize their microbiome composition. The average read depth is 82,082,675 reads (SD = 812,462.4) (Figure S1). Variations in microbiome composition can be appreciated even within the same village. For instance, we observed a pattern of decreasing similarity as individuals live farther away from the village center, at the geographic periphery of the village (Pearson correlation coefficient ρ = 0.311, p = 0.0022, Figures 1B and 1C). In contrast, villagers located at the network center of the social network within each village have a more similar microbiome to the rest of the village, unlike those at the social periphery (linear regression $\beta = 3.66 \times 10^{-5}$, p = 0.761; see STAR Methods for details and also the inset of Figure 1A).

Species, phenotypes, and factors

Overall, we found 2,148 significant associations when looking at 639 microbial species and 123 factors (including physical and mental health, medication use, diet, animal exposure, and social and economic measurements; see Table S1). All comparisons involved appropriate statistical controls (see STAR Methods) and were corrected for multiple hypothesis testing using a false discovery rate procedure. Distinctly, we also found 988 associations with pathways (see Table S2).

The 123 factors are variously measured as continuous and discrete variables (Tables S3, S4, and S5), and, as expected, several of the variables were found to be correlated (for example, individuals with high hemoglobin A1c strongly correlated with reporting a diagnosis of diabetes, and the household wealth index correlated with owning a TV [Figure S2]). Similarly, the clustering of factors based on species effect sizes (obtained from the species-phenotype association models) showed that multiple factors within different categories have similar microbial signatures (Figure S3). Apart from individual phenotypes and factors, broader sub-categories of factors are correlated as well, like diet and economic factors, physiological variables and medication use, education and social factors, and so on. Food and animal factors also have a relative stronger correlation with socioeconomic factors (Figure S2). Overall, this suggests that

CellPress
OPEN ACCESS

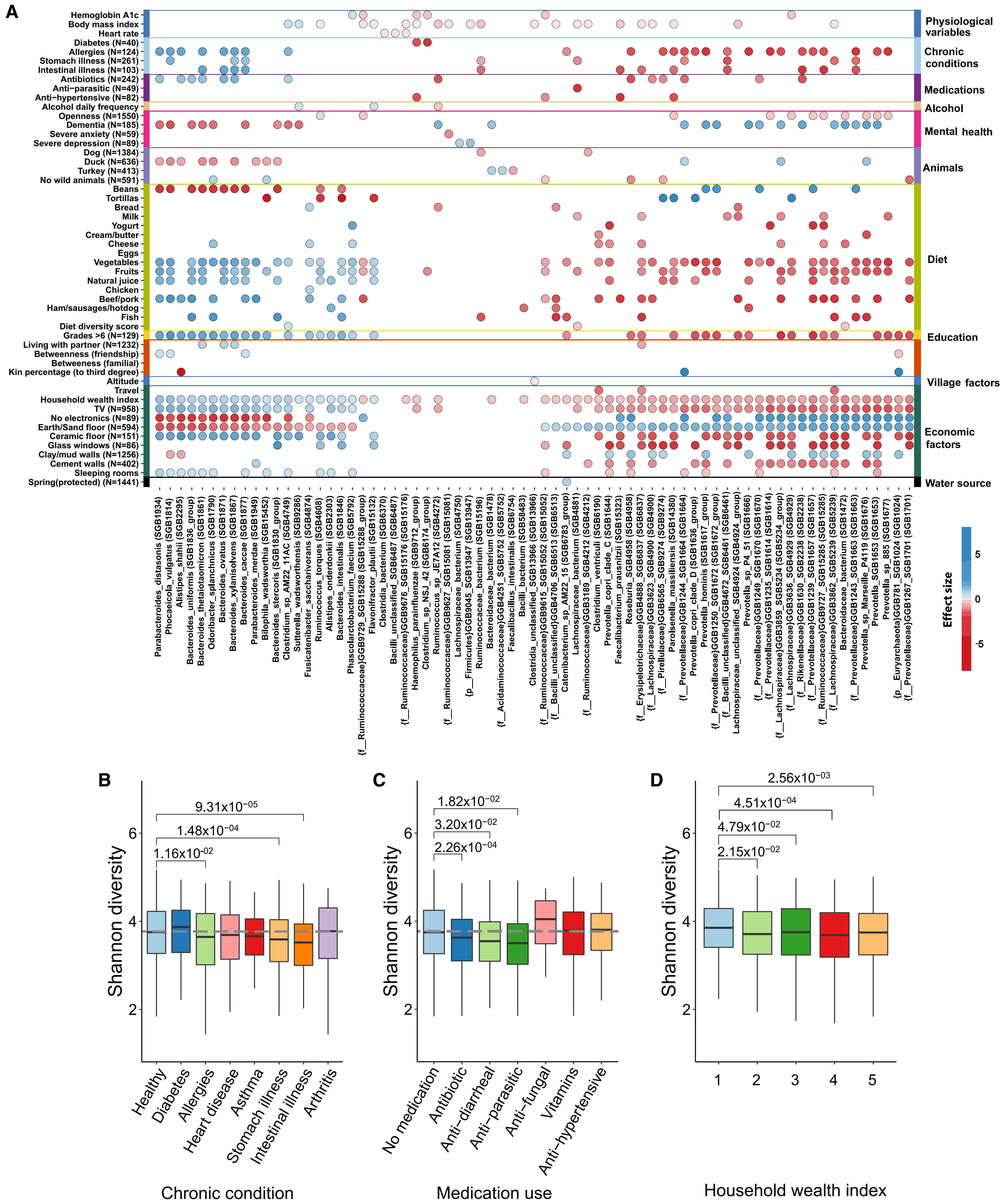

Figure 2. Microbiome association with factors

(A) 81 species that best represent gut microbiome associations with 52 factors (chosen from health, food and animal, and socioeconomic categories; see Table S1 for a complete list of associations). The number of individuals manifesting the respective factor is shown in brackets. The presence of color shows

(legend continued on next page)

economic factors are intertwined with a broad array of factors (like health, food, animal, and other environmental variables), making them even more germane in the context of the gut microbiome in non-Western settings.

Health phenotypes

We found a total of 402 species to be significantly associated with at least one health phenotype (Tables S1 and S3). Among the 402 significant species, 302 of them belonged to the phylum Firmicutes, making it the most associated with health phenotypes. Among all the associated species, 34.58% were identified as unknown[9] at several taxonomic levels. Species uSGB2239 from the Rikenellaceae family and *Parolsenella massiliensis* were the most frequently associated species, significantly associated with 5 health phenotypes; in particular, both were identified as negatively associated with body mass index (BMI), allergies, and intestinal illness (Figure 2A). uSGB2239 was also negatively associated with antibiotics and positively associated with dementia, and *Parolsenella massiliensis* was also associated with anti-hypertensive medication (negatively) and openness (negatively) (see STAR Methods). Microbial species from the Rikenellaceae family have been previously found to be associated with at least one mental health disorder (positively associated with obsessive-compulsive disorder)[10] and enriched in type 2 diabetics in a Pakistani cohort.[11] In another study, Rikenellaceae was found to be significantly associated with high blood sugar in Indian men.[12] Coincidentally, we found that BMI was significantly associated with uSGB2239 of the Rikenellaceae family.

Furthermore, a total of 136 pathways were associated with at least one health phenotype, totaling 157 pathway associations. Among the 157 associations, physiological variables had 85 associations, followed by 24 associations in chronic illness phenotypes, 26 in medication, 2 in acute disease, and 19 in personality measures, alcohol, cigarettes, and mental health (Table S2).

We performed association analysis for a subset of individuals falling in unhealthy ranges of various health phenotypes (i.e., BMI <18 and BMI >25 to account for underweight and overweight individuals, respectively, or diastolic pressure >89 to account for hypertensive individuals) compared to healthy individuals (Figure S4; Table S6). A total of 73 species were associated with multiple unhealthy phenotypes, of which uSGB14313 of the Clostridia family was associated with 3 phenotypes in unhealthy ranges (hemoglobin A1c [5.7–6.4], BMI [25–30], and BMI [30–35]) (Figure S4A; Table S6).

Moving on from individual species, the diversity of an individual's microbiome (measured with Shannon diversity) was computed, with an average alpha diversity of 3.7; incidentally, there was no significant difference in village-level alpha diversity (ANOVA $p = 0.218$). We evaluated whether the alpha diversity itself was associated with various health (and other) phenotypes. The majority of the villagers self-reported themselves as healthy ($n = 1,407$, 75.20%), and only 162 villagers (8.65%) reported having more than one disease. We observed that villagers with reported illnesses (except arthritis and diabetes) had lower diversity relative to healthy villagers (Figure 2B); in particular, villagers with reported stomach (Wilcoxon rank-sum test $p = 1.48 \times 10^{-4}$) and intestinal illnesses (Wilcoxon rank-sum test $p = 9.31 \times 10^{-5}$) had decreased diversity. Villagers who reported taking various medications also had lower diversity (Figure 2C); anti-parasitic drug users showed the lowest diversity (Wilcoxon rank-sum test $p = 0.018$), followed by anti-diarrheal users (Wilcoxon rank-sum test $p = 0.032$) and antibiotic users (Wilcoxon rank-sum test $p = 2.26 \times 10^{-4}$). We found no material associations of microbiome diversity with other categories of medications.

We also performed a contrast analysis by comparing the gut microbiome composition of these self-reported healthy individuals to individuals who reported at least one chronic condition by using differential abundance analysis, and we identified a total of 6 species that were differentially abundant between the two groups (Figure S4B; see STAR Methods). *Lachnospiraceae* bacterium (SGB4906) is the sole species found to be enriched in healthy individuals. On the other hand, uSGB1663 and uSGB27424 of the Prevotellaceae family, *Spirochaetia* bacterium, *Coprococcus*, and uSGB6369 of the Clostridia family were found to be enriched in diseased individuals.

Overall, all the health phenotypes put together contribute 5.7% of the total variance explained in microbial species composition (Figure S5; Table S7). Similarly, 11.6% of the variance in pathway composition is relevant to health phenotypes.

Animal exposure and diet factors

We explored possible associations with animal exposure and diet.[1,13–15] An unusual feature of our setting is that more than 90% of villagers reported having exposure to different types of animals, including wild animals, farm animals, and pets, affording possible zoonotic transmission. Overall, for all food and animal factors, 205 species were found to be significantly associated with at least one of the factors, resulting in 437 associations (Figure 2A; Table S4). Among all the associating bacterial species, 27% were unknown. Among the 205 significantly associated species, 122 of them belonged to Firmicutes, making this phylum the most commonly associated with specific animals or food categories. We found 10 pathways associated with exposure to animals as well (Table S2). Animal exposure

significant associations for that phenotype-species pair (false discovery rate < 0.05); the intensity of the color corresponds to the strength of the effect size. Negative associations are indicated by red and positive by blue. Unknown species are indicated with "{}" specifying the taxonomic level at which the species is known. Listed factors without a sample size are reported for the whole sample.

(B) Shannon diversity of healthy and chronically ill individuals highlights differences in overall microbiome diversity; healthy individuals ($n = 1,407$) are chosen as a reference (gray dashed line).

(C) Shannon diversity is calculated between different medication use categories; non-medicated individuals ($n = 1,246$) are chosen as reference (gray dashed line).

(D) Shannon diversity of villagers belonging to households classified by household wealth index ranging from 1 (least wealthy) to 5 (most wealthy).

All comparisons were performed using the Wilcoxon rank-sum test and corrected for multiple hypothesis testing.

contributed to 2.3% of the variation in species composition. We found no difference in overall Shannon diversity in individuals exposed to different animal categories (Figure S6).

Diet has been extensively studied and shown to have a substantial relationship with the gut microbiome.[15–17] We assessed associations with microbial features and food frequency consumption and found 360 significant associations with diet (Figure 2A). *Bacteroides intestinalis* was the most associated species with food, associated with 8 different food types. In the past, *B. intestinalis* has been implicated in the context of dietary fiber as contributing to an increase of xylan utilization in the gut.[18] Even though most of the individuals' daily diet consists of tortillas and beans, we measured diet diversity using the diet diversity score (DDS)[19] (see STAR Methods and Figure S7). We identified a total of 7 significant associations between the DDS and gut microbiome species (Figure 2A).

We also found 235 pathway associations with food factors (Table S2). Looking at significant associations between pathways and food factors, we found that the pathway L-histidine II degradation (PWY-5028) had a strong positive association with consumption of beef and pork. This pathway was also found to be enriched in humans consuming meat in a previous study,[20] as dipeptides containing histidine are the major form of dipeptides in mammalian skeletal muscle.[21] The role of biologically active peptides is highly correlated with consumption of beef (a protein-rich food) and its enrichment via gut microbiota.[22]

Overall, diet was responsible for 1.85% and 2.14% of the variance explained in our sample in species and pathways composition, respectively (Figure S5).

Socioeconomic factors

Overall, we found 1,105 significant associations (51.44% of total associations) with socioeconomic factors. For all socioeconomic factors, 319 species were found to be significantly associated with at least one of the factors (Figure 2A; Table S1). Among all the 319 associated species, 28.8% of them were unknown, and 185 of them belong to Firmicutes, making it again the most associated phylum for socioeconomic factors. Moreover, uSGB5239 of the Lachnospiraceae family is the most-associated species, statistically significantly associated with 14 socioeconomic factors. We also found 586 associations with pathways, with one of them being associated with 9 socioeconomic factors (Table S2).

Socioeconomic factors are relevant to many exposures and personal habits. Higher monthly expenditures are correlated with a better diet and better household essentials such as a refrigerator or paved floor. We observed that most of the bacteria associated with higher monthly expenditures are the same as the ones associated with better diet quality.[23,24]

Although all the participants in our study are considered to be living in poverty, economic status still varied among them and was associated with possessions and diets potentially relevant to the microbiome; overall, the average household wealth index score (ranging from least wealthy [1] to most wealthy [5]) is 3.26 (SD = 1.33). In terms of measures of economic status, both monthly expenditure and travel were associated with the microbiome. Total wealth was also correlated with owning various items (such as a TV or a mobile phone), some of which (e.g., a refrigerator or a stove) might affect food consumption and others of which (such as having glass windows, cement walls, more sleeping rooms, an earthen floor, or a metal roof) might affect microbiome exposures via other routes (Figures 2A and S3). We observed similar patterns of association where a high wealth index was associated with the same bacterial species associated with owning expensive items (like glass windows), and vice versa. The variance explained by economic factors was 4.13% for species and 3.70% for pathways (Figure S5), indicating the relative importance of economic factors in explaining variation in the gut microbiome composition.

With respect to overall microbial diversity, the subjects from the least well-off households had a Shannon index that was higher than that of the subjects from the wealthier households (in the top 4 quintiles) (Figure 2D).

Overall relationship between species and the phenotypes and factors

From the clustering of associations (Figure 2A) and the dendrogram (Figure S3), it can be observed that different factors can be linked together. This link can be visualized through the relationship between the gut microbiome and the factors. For example, species that are enriched in socioeconomic factors (such as TV ownership and household wealth index) show a similar pattern in vegetable, fruit, and meat consumption. Previous studies have found diet diversity to be correlated with food security and wealth in rural settings.[25,26] Overall, health, food, animal, and socioeconomic factors are clustered together, which is also visibly demonstrated through the microbiome-phenotype lens. Multiple factors from different categories can also be tied together by a singles species. For instance, uSGB5239 (of the Lachnospiraceae family) is associated with 22 different phenotypes from the health category (BMI, stomach illness, dementia), the food and animal category (vegetables, fruits, natural juice, beef/pork, fish), and the socioeconomic category (grades >6, travel, household wealth index, TV, no electronics, earth/sand floor, ceramic floor, glass windows, clay/mud walls, cement walls, and sleeping rooms).[27]

Comparison with other datasets and countries

We compared microbial signature across datasets from other countries. Across the nine cohorts considered, we identified BMI to be the sole host phenotype shared across all of them.[6,28–35] In our dataset, we also identified BMI to be one of the phenotypes with the most significant associations ($n = 275$). Therefore, a meta-analysis of BMI on 5,001 samples from the nine cohorts identified 21 significant species. *Sutterella wadsworthensis* (SGB9286) was found to be associated with a higher BMI in most of the datasets, while *Bacilli* bacterium (SGB47359) is the most negatively associated species (Figure 3). Coefficients from our cohort are statistically significant for all 21 species. Among other cohorts, one cohort in particular had the greatest number of significant coefficients (13 out of 21).[28]

Higher abundances of *Parabacteroides merdae* (SGB1949) have been found in obese mice and have also exhibited a protective effect against obesity-associated atherosclerosis.[36] In human studies, *Butyrivibrio crossotus* (SGB5065) was found to be enriched in non-obese individuals,[37] and *Sutterella wadsworthensis* (SGB9286; one of our strongest positively correlated

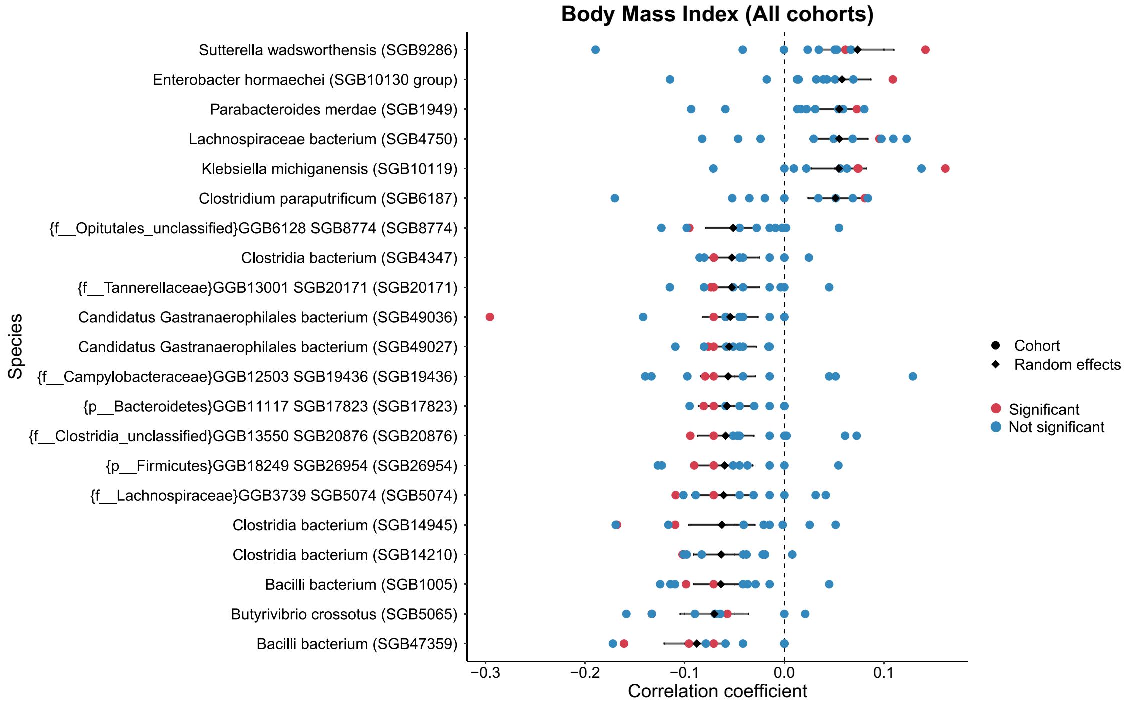

Figure 3. Meta-analysis of bacterial species associated with BMI across different cohorts

Random-effect meta-analysis of BMI (body mass index) on 5,001 gut metagenomes species-level profiles across different Western and non-Western cohorts (points shown indicates correlation coefficients from each separate cohort). Species with statistically significant random effects estimates ($n = 21$) were included (see STAR Methods for more details and Table S11).

species) was found to be 10 times more abundant in obese children-adolescents[38]—both of which were consistent with these meta-analysis findings.

Furthermore, we also compared our associations with the ones found by the Dutch Microbiome Project. We found 13 species-phenotype associations in common with the Dutch study, 8 of which were with BMI, and *Butyrivibrio crossotus*, *Roseburia inulinivorans*, *Faecalibacterium prausnitzii*, *Methanobrevibacter smithii*, *Eubacterium siraeum*, *Haemophilus parainfluenzae*, *Mitsuokella multacida*, and *Flavonifractor plautii* were found to be significantly associated in both datasets with BMI. Moreover, *Haemophilus parainfluenzae* was also significant for hemoglobin A1c in both datasets. *Ruminococcus torques* was significant in both datasets for antibiotic use. Finally, monthly income/expenditure had 3 significant species in common: *Alistipes shahii*, *Barnesiella intestinihominis*, and *Flavonifractor plautii*. The presence of very few significant species in common between the Honduras and Netherlands cohorts is largely due to differences in measurements between the two cohorts and the relatively low number of common species across the two cohorts (see Table S8 for a list of possible comparisons).

Relevance of microbial strains

Finally, moving beyond species-specific associations with phenotypes and factors, we observed a meaningful variation between the genetic makeup of the same species across different individuals that is, in turn, associated with diverse factors (Figure 4A). For instance, individuals with a higher household wealth index are likely to have a different strain of *Eubacterium rectale* compared to less wealthy individuals in a set of 1,610 individuals (Fisher's exact test $p = 4.99 \times 10^{-4}$) (Figure 4A).

Moreover, adding strain-phylogenetic information in the model alters the relationship between species and factors overall (Figure 4B) by inducing a small shift. Among all the effect sizes, 0.2% of them switch direction when adding the phylogenetic effect (Figure S8; Table S9).

Looking deeper into the strain diversity in individuals, we evaulated the variation of the percentage of polymorphic sites across individuals and factors. As an illustration, we observed that wealthier individuals ($\beta = 0.08345$, $p = 1.26 \times 10^{-7}$) or those consuming a higher number of eggs ($\beta = 0.626$, $p = 0.1146$) had a higher percentage of polymorphic sites (Figures 5A and 5B; Table S10). This comports with findings in another study where percentages of polymorphic sites from just *Prevotella copri* strains were found to be different between recent South Asian Canadian immigrants and first-generation South Asian Canadians.[39]

DISCUSSION

Integrated, standardized, large, population-based cohorts to study the microbiome are uncommon, but such studies offer the prospect of identifying factors shaping the gut microbiome or being shaped by it. By extending our knowledge of the human gut microbiome to a novel population in a lower-and-middle-income (LMIC) setting, assessing previously uncharacterized taxa,

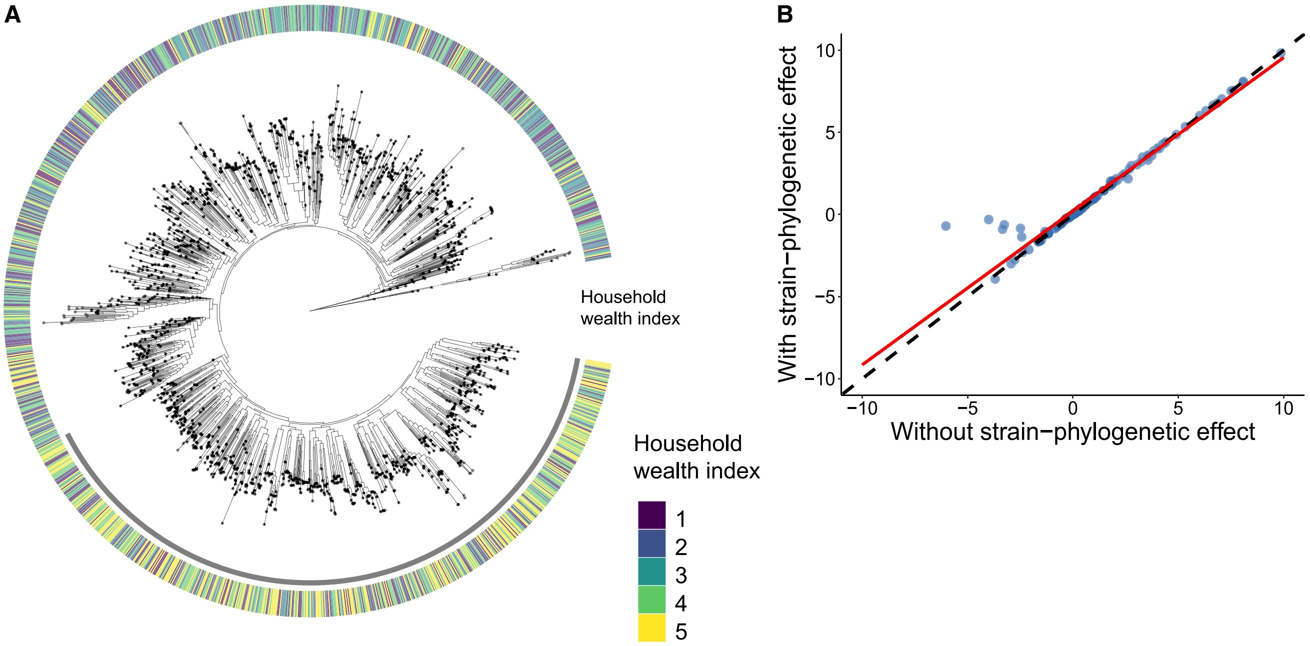

Figure 4. Microbial strain association with host factors

(A) In this strain-level phylogeny of *Eubacterium rectale* (SGB4933 group) (as an illustrative microbe) in 1,610 individuals, leaves are annotated with the household wealth index (as an illustrative host factor). A cluster of individuals (annotated in gray) situated on a different strain of *Eubacterium rectale* (separated branch) are more likely to have higher wealth compared to rest of the individuals (Fisher's exact test $p = 0.0004998$).

(B) Comparison of significant effect sizes obtained from a linear mixed model with and without adding strain-level phylogeny information across significant species-phenotype relationships overall. Coefficients from the association models with and without phylogenetic information are positively correlated (Spearman correlation coefficient $\rho = 0.989$, $p < 2.2 \times 10^{-16}$), and the red line is the linear fit ($\beta = 0.9351$, intercept = 0.2016, $p < 2.2 \times 10^{-16}$), showing the relationship between the two models. The deviation of the red fitted line from the dashed line shows the important effect of adding the strain-level phylogeny in the species-phenotype association model (Table S9).

having a very broad range of phenotypes and factors, and using strain-level genomic information, our goal is to advance understanding of the possible relationship of the gut microbiome with diverse human attributes.

We find that variation in the gut microbiome across individuals living in a traditional way in remote Honduran villages is partly explained by variations in diet, lifestyle, environment, and health factors. Overall, we found 2,148 unique associations between 639 bacterial species and 123 phenotypes/factors. Examining pairwise correlations between phenotypes and all gut microbial species, broader categories like food and animal factors were highly associated with socioeconomic factors, suggesting that wealth is an important underlying factor in this non-Western cohort. We also observe diet to be highly correlated with education and social factors. The associations between species and attributes included many uncharacterized species, which in many cases were shown to have a stronger effect than known species. Phenotype and factor associations were also identified after accounting for strain-level phylogenies, which often had a profound effect on the extent of the association between microbiome species and the attributes under consideration.

Still, despite measuring a large number and variety of factors, only 19.2% of the variation across individuals in microbiome composition was accounted for by these factors, in keeping with prior studies.[1,40–42] This suggests that microbiome composition in individuals may be quite idiosyncratic or may depend on details of social interactions or unmeasured environmental exposures. Rare species may also help account for this variation. The current understanding of how individual and population-level microbiomes come to be shaped is thus still incomplete. Nevertheless, the factors we ascertained in Honduras did combine to account for 19.2% of the species variation (as noted) and 33.4% of the pathway variation; this may be compared to a study from the Netherlands where the measured phenotypes accounted for 13% and 16.2% of the variation, respectively,[1] although different methodologies for taxonomic and functional characterization were used here, reflecting ongoing methodological advances. Shotgun metagenomic sequencing enabled us to further classify the functionality aspects of the gut microbiome, giving us a distinct advantage over 16S rRNA sequencing. Accurate profiling of the microbiome can be impacted by the choice of primers in the 16S method.[43] Furthermore, in addition to these limitations to the 16S method, updated reference databases and tools enable us to profile a far greater number of species.[6,44]

It has already been established that the gut microbiome composition can be related to various health conditions in both humans and mice,[45] and conditions like cancer, obesity, diabetes, anxiety, and depression can induce shifts in gut composition (as previously shown in many mostly Western

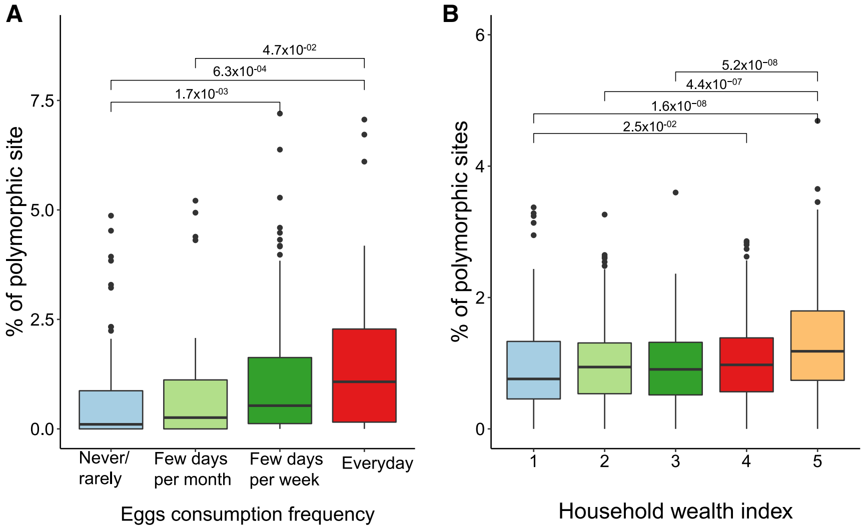

Figure 5. Relationship of variability in polymorphic sites with two host factors

(A) Polymorphic site variability of uSGB4905 shows a gradual increase in the percentage of polymorphic sites in individuals consuming more eggs in their regular diet ($n = 312$).

(B) Another demonstration of how variability in polymorphic sites changes with phenotype. Here, progressively wealthier individuals have a higher percentage of polymorphic sites in *Prevotella copri clade C* (SGB1644) ($n = 1,458$).

populations).[1,45–51] Alcohol intake and cigarette use have been linked to gut microbiome dysbiosis, as well as medications.[52–56] In keeping with these prior studies, we confirm such findings in this rural LMIC cohort.[3–5,57] Indeed, we found 606 associations between the microbiome and health-related phenotypes and factors. Chronic illnesses and medication use were the most strongly associated. Among chronic illnesses, intestinal illnesses show the greatest differences. We uncovered 273 total associations between gut microbiome species and physiological measurement ranges that may be linked to underlying chronic conditions such as obesity, diabetes, and hypertension. Moreover, we found 62 associations with mental health phenotypes alone, a relatively understudied area.

Comparisons with other non-Western cohorts can highlight some of the differences and similarities between Honduras and such cohorts as well. Comparing BMIs across 5 other non-Western cohorts (from India, Cameroon [2], Peru, and Madagascar) and 4 western cohorts (Great Britain/USA, USA [2], and China) using a meta-analysis approach, we found 21 species significantly associated with BMI in all cohorts, among which the Honduran correlation coefficients were consistently significant and close to the unbiased estimate, reflecting stable yet significant associations because of the sample size.

Looking at the overall microbial composition among healthy and chronically ill subjects, the Shannon diversity was generally lower in chronically ill people, especially those with allergies and gastrointestinal illnesses. Moreover, comparing healthy individuals to those who are chronically ill, we found 6 taxa to be differentially enriched in one of the groups. *Lachnospiraceae* bacterium is the only significant species differentially enriched in healthy individuals. On the other hand, 2 unknown and 3 known species, uSGB1663 and uSGB27424 of the Prevotellaceae family, *Spirochaetia* bacterium, *Coprococcus*, and uSGB6369 of the Clostridia family, were differentially enriched in chronically diseased individuals. Among medication users, those taking anti-parasitic medication had the largest drop in overall diversity.

Another factor that greatly influences the gut microbiome is diet. Our sample population exhibits a consistent diet, with beans and tortillas consumed by most people daily. Still, we found 360 associations with food categories. Moreover, just as a previously studied Dutch cohort found that pets had notable associations with the microbiome,[1] we likewise found 77 associations with a (broader) range of animal exposures.

Social and economic factors had 1,105 associations, with the bulk of the strong associations coming from unknown species. The gut microbiome samples had 947 unique associations with economic factors alone, making it the second highest associated category of variables we examined, after health. Wealth differences in individuals can also manifest in the form of more diverse strains in some species being present in wealthier individuals. Prior research in Honduras has highlighted the crucial importance of socioeconomic status in addressing health in such communities,[58] and the microbiome varies in important ways along this axis, even in this poor setting.

Social interaction is an integral part of Honduran villagers' life. In total, 123 unique associations with various social network factors were found. Studies investigating social interactions between mice have shown the evolutionary advantage of having behaviors that enhance social interaction that consequently facilitates microbiome transmission.[45,59,60] In wild mice, social associations are predictive of microbiome composition, and the microbiome is correlated across mice interaction networks.[61] In humans, strain-level similarities have been shown in familial and partner networks within and outside households.[62–64] Whether these interactions translate into exposures that directly contribute to health is an important area for further work.

Finally, our samples were collected from individuals spread across 19 villages separated in space and elevation, and the overall gut microbiome samples were observed to vary with the relative spatial position within the villages; the dissimilarity score with a village-averaged microbiome increased as subjects lived further away from the village center. Relatedly, we found 3 significant associations with elevation.

Uncharacterized taxa play a vital role in all these associations, as in prior LMIC cohorts.[6] Despite the number of unknown species in the Honduran cohort being about a third of total species, their relative strength of associations was observed to be higher in all the phenotype/factor categories. Distinctly, strain-level

CellPress
OPEN ACCESS

information is also relevant to the microbiome-phenotype relationship and should, optimally, be accounted for.

Limitations of the study

To fully understand the effects of host factors like diet and environment, surveying the exact quantity of various food groups (in addition to the food frequency questionnaire) could improve the accuracy of our associations of diet with gut microbiome. As for environmental factors, investigating the exact sources of water and food shared between individuals could help more accurately answer the impact of a shared environment on the gut microbiome in comparison to other host factors. And, of course, our findings arise from the analysis of data from a single region of the world.

Conclusions

These findings advance understanding the interplay between various phenotypes and host factors on the one hand, and the gut microbiome on the other. By expanding our knowledge of the human microbiome to a novel non-Western cohort, it is possible to further our understanding of the role of the gut microbiome in chronic illness and, at the same time, open up opportunities to use such findings to develop inexpensive biomarkers to aid diagnostics in rural settings.[65–67] To the extent that a healthy microbiome is driven by modifiable social and environmental factors (such as diet, smoking, living arrangements, lifestyle, and so on), understanding which factors to target or what possible microbiome-modifying interventions to implement could help advance individual and collective health in diverse settings.

STAR★METHODS

Detailed methods are provided in the online version of this paper and include the following:

- KEY RESOURCES TABLE
- RESOURCE AVAILABILITY
  - Lead contact
  - Materials availability
  - Data and code availability
- EXPERIMENT MODEL AND STUDY PARTICIPATION DETAILS
  - Sample collection, library preparation, and sequencing
  - Local involvement in the research
- METHOD DETAILS
  - Taxonomic profiling and diversity analysis
  - Factor characterization
  - Population-weighted village centroid
- QUANTIFICATION AND STATISTICAL ANALYSIS
  - Model for microbiome-factor regression
  - Meta-analysis of BMI across non-Western cohorts
  - Strain-factor analysis and phylogenetic signal
  - Polymorphic sites analysis
  - Differential abundance analysis

SUPPLEMENTAL INFORMATION

Supplemental information can be found online at https://doi.org/10.1016/j.celrep.2024.114442.

ACKNOWLEDGMENTS

We thank all the study participants in Honduras. We thank Jose Eduardo Gámez for coordinating the fieldwork; Rennie Negron, Liza Nicoll, and Thomas Keegan for their support on field operations, data collection, and administrative support; YCGA (Yale Center for Genomic Analysis) for sequencing the metagenomic libraries (NIH award 1S10OD030363-01A1); and Qiaojuan Shi for processing the specimens and handling the extractions. We thank Michael Baym and Mark Gerstein for helpful comments on the manuscript. This work was supported by the NOMIS Foundation, with additional support from Schmidt Futures, the Pershing Square Foundation, and the Rothberg Catalyzer Fund. Empanelment of the underlying cohort was supported by the Bill and Melinda Gates Foundation.

AUTHOR CONTRIBUTIONS

Conceptualization, S.V.S., F.B., M.A., I.L.B., and N.A.C.; methodology, S.V.S., F.B., M.A., A.S., I.L.B., and N.A.C.; data collection, S.V.S., F.B., M.A., R.M.J., I.L.B., and N.A.C.; statistical analysis, S.V.S., F.B., and M.A.; funding acquisition, N.A.C.; supervision, I.L.B. and N.A.C.; writing, S.V.S., F.B., M.A., I.L.B., and N.A.C.

DECLARATION OF INTERESTS

The authors declare no competing interests.

Received: January 9, 2024
Revised: April 27, 2024
Accepted: June 19, 2024
Published: July 4, 2024

REFERENCES

1. Gacesa, R., Kurilshikov, A., Vich Vila, A., Sinha, T., Klaassen, M.A.Y., Bolte, L.A., Andreu-Sánchez, S., Chen, L., Collij, V., Hu, S., et al. (2022). Environmental factors shaping the gut microbiome in a Dutch population. Nature *604*, 732–739.
2. Abdill, R.J., Adamowicz, E.M., and Blekhman, R. (2022). Public human microbiome data are dominated by highly developed countries. PLoS Biol. *20*, e3001536.
3. Mohanan, M., Vera-Hernández, M., Das, V., Giardili, S., Goldhaber-Fiebert, J.D., Rabin, T.L., Raj, S.S., Schwartz, J.I., and Seth, A. (2015). The know-do gap in quality of health care for childhood diarrhea and pneumonia in rural India. JAMA Pediatr. *169*, 349–357.
4. Young, B.N., Clark, M.L., Rajkumar, S., Benka-Coker, M.L., Bachand, A., Brook, R.D., Nelson, T.L., Volckens, J., Reynolds, S.J., L'Orange, C., et al. (2019). Exposure to household air pollution from biomass cookstoves and blood pressure among women in rural Honduras: A cross-sectional study. Indoor Air *29*, 130–142.
5. Hartley, D. (2004). Rural health disparities, population health, and rural culture. Am. J. Public Health *94*, 1675–1678.
6. Pasolli, E., Asnicar, F., Manara, S., Zolfo, M., Karcher, N., Armanini, F., Beghini, F., Manghi, P., Tett, A., Ghensi, P., et al. (2019). Extensive Unexplored Human Microbiome Diversity Revealed by Over 150,000 Genomes from Metagenomes Spanning Age, Geography, and Lifestyle. Cell *176*, 649–662.e20.
7. Shakya, H.B., Stafford, D., Hughes, D.A., Keegan, T., Negron, R., Broome, J., McKnight, M., Nicoll, L., Nelson, J., Iriarte, E., et al. (2017). Exploiting social influence to magnify population-level behaviour change in maternal and child health: study protocol for a randomised controlled trial of network targeting algorithms in rural Honduras. BMJ Open *7*, e012996.
8. Airoldi, E., and Christakis, N.A. (2024). Induction of Social Contagion Across Diverse Outcomes in Structured Experiments in Isolated Honduras Villages. Science *384*, eadi5147.
9. Blanco-Míguez, A., Beghini, F., Cumbo, F., McIver, L.J., Thompson, K.N., Zolfo, M., Manghi, P., Dubois, L., Huang, K.D., Thomas, A.M., et al. (2023). Extending and improving metagenomic taxonomic profiling with uncharacterized species using MetaPhlAn 4. Nat. Biotechnol. *41*, 1633–1644. https://doi.org/10.1038/s41587-023-01688-w.

10. Domènech, L., Willis, J., Alemany-Navarro, M., Morell, M., Real, E., Escaramís, G., Bertolín, S., Sánchez Chinchilla, D., Balcells, S., Segalàs, C., et al. (2022). Changes in the stool and oropharyngeal microbiome in obsessive-compulsive disorder. Sci. Rep. *12*, 1448.

11. Saleem, A., Ikram, A., Dikareva, E., Lahtinen, E., Matharu, D., Pajari, A.-M., de Vos, W.M., Hasan, F., Salonen, A., and Jian, C. (2022). Unique Pakistani gut microbiota highlights population-specific microbiota signatures of type 2 diabetes mellitus. Gut Microb. *14*, 2142009.

12. Singh, K.S., Paul, D., Gupta, A., Dhotre, D., Klawonn, F., and Shouche, Y. (2023). Indian sewage microbiome has unique community characteristics and potential for population-level disease predictions. Sci. Total Environ. *858*, 160178.

13. Balakumar, M., Prabhu, D., Sathishkumar, C., Prabu, P., Rokana, N., Kumar, R., Raghavan, S., Soundarajan, A., Grover, S., Batish, V.K., et al. (2018). Improvement in glucose tolerance and insulin sensitivity by probiotic strains of Indian gut origin in high-fat diet-fed C57BL/6J mice. Eur. J. Nutr. *57*, 279–295.

14. Baxter, N.T., Schmidt, A.W., Venkataraman, A., Kim, K.S., Waldron, C., and Schmidt, T.M. (2019). Dynamics of Human Gut Microbiota and Short-Chain Fatty Acids in Response to Dietary Interventions with Three Fermentable Fibers. mBio *10*, e02566-18. https://doi.org/10.1128/mBio.02566-18.

15. Bokulich, N.A., Chung, J., Battaglia, T., Henderson, N., Jay, M., Li, H., D Lieber, A., Wu, F., Perez-Perez, G.I., Chen, Y., et al. (2016). Antibiotics, birth mode, and diet shape microbiome maturation during early life. Sci. Transl. Med. *8*, 343ra82.

16. Baumann-Dudenhoeffer, A.M., D'Souza, A.W., Tarr, P.I., Warner, B.B., and Dantas, G. (2018). Infant diet and maternal gestational weight gain predict early metabolic maturation of gut microbiomes. Nat. Med. *24*, 1822–1829.

17. Bonaccio, M., Di Castelnuovo, A., Bonanni, A., Costanzo, S., De Lucia, F., Pounis, G., Zito, F., Donati, M.B., de Gaetano, G., and Iacoviello, L.; Moli-sani project Investigators* (2013). Adherence to a Mediterranean diet is associated with a better health-related quality of life: a possible role of high dietary antioxidant content. BMJ Open *3*, e003003. https://doi.org/10.1136/bmjopen-2013-003003.

18. Hong, P.-Y., Iakiviak, M., Dodd, D., Zhang, M., Mackie, R.I., and Cann, I. (2014). Two new xylanases with different substrate specificities from the human gut bacterium Bacteroides intestinalis DSM 17393. Appl. Environ. Microbiol. *80*, 2084–2093.

19. Kim, S., Haines, P.S., Siega-Riz, A.M., and Popkin, B.M. (2003). The Diet Quality Index-International (DQI-I) provides an effective tool for cross-national comparison of diet quality as illustrated by China and the United States. J. Nutr. *133*, 3476–3484.

20. Abe, H., Okuma, E., Sekine, H., Maeda, A., and Yoshiue, S. (1993). Human urinary excretion of L-histidine-related compounds after ingestion of several meats and fish muscle. Int. J. Biochem. *25*, 1245–1249.

21. Boldyrev, A.A., and Severin, S.E. (1990). The histidine-containing dipeptides, carnosine and anserine: distribution, properties and biological significance. Adv. Enzyme Regul. *30*, 175–194.

22. Albracht-Schulte, K., Islam, T., Johnson, P., and Moustaid-Moussa, N. (2021). Systematic Review of beef protein effects on gut Microbiota: Implications for health. Adv. Nutr. *12*, 102–114.

23. Darmon, N., and Drewnowski, A. (2015). Contribution of food prices and diet cost to socioeconomic disparities in diet quality and health: a systematic review and analysis. Nutr. Rev. *73*, 643–660.

24. Darmon, N., Ferguson, E.L., and Briend, A. (2002). A cost constraint alone has adverse effects on food selection and nutrient density: an analysis of human diets by linear programming. J. Nutr. *132*, 3764–3771.

25. Rammohan, A., Pritchard, B., and Dibley, M. (2019). Home gardens as a predictor of enhanced dietary diversity and food security in rural Myanmar. BMC Publ. Health *19*, 1145.

26. Ali, N.B., Tahsina, T., Hoque, D.M.E., Hasan, M.M., Iqbal, A., Huda, T.M., and El Arifeen, S. (2019). Association of food security and other socio-economic factors with dietary diversity and nutritional statuses of children aged 6-59 months in rural Bangladesh. PLoS One *14*, e0221929.

27. Tang, M., Weaver, N.E., Frank, D.N., Ir, D., Robertson, C.E., Kemp, J.F., Westcott, J., Shankar, K., Garces, A.L., Figueroa, L., et al. (2022). Longitudinal reduction in diversity of maternal gut Microbiota during pregnancy is observed in multiple low-resource settings: Results from the Women First trial. Front. Microbiol. *13*, 823757.

28. Asnicar, F., Berry, S.E., Valdes, A.M., Nguyen, L.H., Piccinno, G., Drew, D.A., Leeming, E., Gibson, R., Le Roy, C., Khatib, H.A., et al. (2021). Microbiome connections with host metabolism and habitual diet from 1,098 deeply phenotyped individuals. Nat. Med. *27*, 321–332.

29. Kaur, K., Khatri, I., Akhtar, A., Subramanian, S., and Ramya, T.N.C. (2020). Metagenomics analysis reveals features unique to Indian distal gut microbiota. PLoS One *15*, e0231197.

30. Lokmer, A., Cian, A., Froment, A., Gantois, N., Viscogliosi, E., Chabé, M., and Ségurel, L. (2019). Use of shotgun metagenomics for the identification of protozoa in the gut microbiota of healthy individuals from worldwide populations with various industrialization levels. PLoS One *14*, e0211139.

31. Human Microbiome Project Consortium (2012). Structure, function and diversity of the healthy human microbiome. Nature *486*, 207–214.

32. Integrative, H.M.P.; iHMP) Research Network Consortium (2019). The Integrative Human Microbiome Project. Nature *569*, 641–648.

33. Qin, N., Yang, F., Li, A., Prifti, E., Chen, Y., Shao, L., Guo, J., Le Chatelier, E., Yao, J., Wu, L., et al. (2014). Alterations of the human gut microbiome in liver cirrhosis. Nature *513*, 59–64.

34. Obregon-Tito, A.J., Tito, R.Y., Metcalf, J., Sankaranarayanan, K., Clemente, J.C., Ursell, L.K., Zech Xu, Z., Van Treuren, W., Knight, R., Gaffney, P.M., et al. (2015). Subsistence strategies in traditional societies distinguish gut microbiomes. Nat. Commun. *6*, 6505.

35. Rubel, M.A., Abbas, A., Taylor, L.J., Connell, A., Tanes, C., Bittinger, K., Ndze, V.N., Fonsah, J.Y., Ngwang, E., Essiane, A., et al. (2020). Lifestyle and the presence of helminths is associated with gut microbiome composition in Cameroonians. Genome Biol. *21*, 122.

36. Qiao, S., Liu, C., Sun, L., Wang, T., Dai, H., Wang, K., Bao, L., Li, H., Wang, W., Liu, S.-J., and Liu, H. (2023). Publisher Correction: Gut Parabacteroides merdae protects against cardiovascular damage by enhancing branched-chain amino acid catabolism. Nat. Metab. *5*, 184.

37. Meijnikman, A.S., Aydin, O., Prodan, A., Tremaroli, V., Herrema, H., Levin, E., Acherman, Y., Bruin, S., Gerdes, V.E., Backhed, F., et al. (2020). Distinct differences in gut microbial composition and functional potential from lean to morbidly obese subjects. J. Intern. Med. *288*, 699–710.

38. Squillario, M., Bonaretti, C., La Valle, A., Di Marco, E., Piccolo, G., Minuto, N., Patti, G., Napoli, F., Bassi, M., Maghnie, M., et al. (2023). Gut-microbiota in children and adolescents with obesity: inferred functional analysis and machine-learning algorithms to classify microorganisms. Sci. Rep. *13*, 11294.

39. Copeland, J.K., Chao, G., Vanderhout, S., Acton, E., Wang, P.W., Benchimol, E.I., El Sohami, A., Croitoru, K., Gommerman, J.L., and Guttman, D.S.; GEMINI Research Team (2021). The impact of migration on the gut metagenome of south Asian Canadians. Gut Microb. *13*, 1–29.

40. Falony, G., Joossens, M., Vieira-Silva, S., Wang, J., Darzi, Y., Faust, K., Kurilshikov, A., Bonder, M.J., Valles-Colomer, M., Vandeputte, D., et al. (2016). Population-level analysis of gut microbiome variation. Science *352*, 560–564.

41. Blacher, E., Bashiardes, S., Shapiro, H., Rothschild, D., Mor, U., Dori-Bachash, M., Kleimeyer, C., Moresi, C., Harnik, Y., Zur, M., et al. (2019). Potential roles of gut microbiome and metabolites in modulating ALS in mice. Nature *572*, 474–480.

42. Manor, O., Dai, C.L., Kornilov, S.A., Smith, B., Price, N.D., Lovejoy, J.C., Gibbons, S.M., and Magis, A.T. (2020). Health and disease markers

correlate with gut microbiome composition across thousands of people. Nat. Commun. *11*, 5206–5212.

43. Tremblay, J., Singh, K., Fern, A., Kirton, E.S., He, S., Woyke, T., Lee, J., Chen, F., Dangl, J.L., and Tringe, S.G. (2015). Primer and platform effects on 16S rRNA tag sequencing. Front. Microbiol. *6*, 771.
44. Peterson, D., Bonham, K.S., Rowland, S., Pattanayak, C.W., and RESONANCE Consortium; and Klepac-Ceraj, V. (2021). Comparative analysis of 16S rRNA gene and metagenome sequencing in pediatric gut microbiomes. Front. Microbiol. *12*, 670336.
45. Cryan, J.F., O'Riordan, K.J., Cowan, C.S.M., Sandhu, K.V., Bastiaanssen, T.F.S., Boehme, M., Codagnone, M.G., Cussotto, S., Fulling, C., Golubeva, A.V., et al. (2019). The Microbiota-Gut-Brain Axis. Physiol. Rev. *99*, 1877–2013.
46. Patterson, E., Ryan, P.M., Cryan, J.F., Dinan, T.G., Ross, R.P., Fitzgerald, G.F., and Stanton, C. (2016). Gut microbiota, obesity and diabetes. Postgrad. Med. J. *92*, 286–300.
47. Turnbaugh, P.J., Hamady, M., Yatsunenko, T., Cantarel, B.L., Duncan, A., Ley, R.E., Sogin, M.L., Jones, W.J., Roe, B.A., Affourtit, J.P., et al. (2009). A core gut microbiome in obese and lean twins. Nature *457*, 480–484.
48. Ley, R.E., Turnbaugh, P.J., Klein, S., and Gordon, J.I. (2006). Microbial ecology: human gut microbes associated with obesity. Nature *444*, 1022–1023.
49. Murri, M., Leiva, I., Gomez-Zumaquero, J.M., Tinahones, F.J., Cardona, F., Soriguer, F., and Queipo-Ortuño, M.I. (2013). Gut microbiota in children with type 1 diabetes differs from that in healthy children: a case-control study. BMC Med. *11*, 46.
50. Qin, J., Li, Y., Cai, Z., Li, S., Zhu, J., Zhang, F., Liang, S., Zhang, W., Guan, Y., Shen, D., et al. (2012). A metagenome-wide association study of gut microbiota in type 2 diabetes. Nature *490*, 55–60.
51. Hu, B., Elinav, E., Huber, S., Strowig, T., Hao, L., Hafemann, A., Jin, C., Wunderlich, C., Wunderlich, T., Eisenbarth, S.C., and Flavell, R.A. (2013). Microbiota-induced activation of epithelial IL-6 signaling links inflammasome-driven inflammation with transmissible cancer. Proc. Natl. Acad. Sci. USA *110*, 12852.
52. Sarkola, T., Iles, M.R., Kohlenberg-Mueller, K., and Eriksson, C.J.P. (2002). Ethanol, acetaldehyde, acetate, and lactate levels after alcohol intake in white men and women: effect of 4-methylpyrazole. Alcohol Clin. Exp. Res. *26*, 239–245.
53. Allais, L., Kerckhof, F.-M., Verschuere, S., Bracke, K.R., De Smet, R., Laukens, D., Van den Abbeele, P., De Vos, M., Boon, N., Brusselle, G.G., et al. (2016). Chronic cigarette smoke exposure induces microbial and inflammatory shifts and mucin changes in the murine gut. Environ. Microbiol. *18*, 1352–1363.
54. Botschuijver, S., Roeselers, G., Levin, E., Jonkers, D.M., Welting, O., Heinsbroek, S.E.M., de Weerd, H.H., Boekhout, T., Fornai, M., Masclee, A.A., et al. (2017). Intestinal Fungal Dysbiosis Is Associated With Visceral Hypersensitivity in Patients With Irritable Bowel Syndrome and Rats. Gastroenterology *153*, 1026–1039.
55. Caputi, V., Marsilio, I., Filpa, V., Cerantola, S., Orso, G., Bistoletti, M., Paccagnella, N., De Martin, S., Montopoli, M., Dall'Acqua, S., et al. (2017). Antibiotic-induced dysbiosis of the microbiota impairs gut neuromuscular function in juvenile mice. Br. J. Pharmacol. *174*, 3623–3639.
56. Fröhlich, E.E., Farzi, A., Mayerhofer, R., Reichmann, F., Jačan, A., Wagner, B., Zinser, E., Bordag, N., Magnes, C., Fröhlich, E., et al. (2016). Cognitive impairment by antibiotic-induced gut dysbiosis: Analysis of gut microbiota-brain communication. Brain Behav. Immun. *56*, 140–155.
57. Kim, D.A., Hwong, A.R., Stafford, D., Hughes, D.A., O'Malley, A.J., Fowler, J.H., and Christakis, N.A. (2015). Social network targeting to maximise population behaviour change: a cluster randomised controlled trial. Lancet *386*, 145–153.
58. Arps, S. (2011). Socioeconomic status and body size among women in Honduran Miskito communities. Ann. Hum. Biol. *38*, 508–519.
59. Rosenberg, E., Sharon, G., and Zilber-Rosenberg, I. (2009). The hologenome theory of evolution contains Lamarckian aspects within a Darwinian framework. Environ. Microbiol. *11*, 2959–2962.
60. Carlson, S.J., O'Loughlin, A.A., Anez-Bustillos, L., Baker, M.A., Andrews, N.A., Gunner, G., Dao, D.T., Pan, A., Nandivada, P., Chang, M., et al. (2019). A Diet With Docosahexaenoic and Arachidonic Acids as the Sole Source of Polyunsaturated Fatty Acids Is Sufficient to Support Visual, Cognitive, Motor, and Social Development in Mice. Front. Neurosci. *13*, 72.
61. Raulo, A., Allen, B.E., Troitsky, T., Husby, A., Firth, J.A., Coulson, T., and Knowles, S.C.L. (2021). Social networks strongly predict the gut microbiota of wild mice. ISME J. *15*, 2601–2613.
62. Brito, I.L., Gurry, T., Zhao, S., Huang, K., Young, S.K., Shea, T.P., Naisilisili, W., Jenkins, A.P., Jupiter, S.D., Gevers, D., and Alm, E.J. (2019). Transmission of human-associated microbiota along family and social networks. Nat. Microbiol. *4*, 964–971.
63. Valles-Colomer, M., Blanco-Míguez, A., Manghi, P., Asnicar, F., Dubois, L., Golzato, D., Armanini, F., Cumbo, F., Huang, K.D., Manara, S., et al. (2023). The person-to-person transmission landscape of the gut and oral microbiomes. Nature *614*, 125–135.
64. Pullman, J., Beghini, F., Alexander, M., Shridhar, S.V., Prinster, D., Brito, I.L., and Christakis, N.A. (2023). Detailed social network interactions and gut microbiome strain-sharing within isolated Honduras villages. Preprint at bioRxiv. https://doi.org/10.1101/2023.04.06.535875.
65. Xiao, L., Zhang, F., and Zhao, F. (2022). Large-scale microbiome data integration enables robust biomarker identification. Nat. Comput. Sci. *2*, 307–316.
66. Wynford-Thomas, R., and Robertson, N.P. (2017). The economic burden of chronic neurological disease. J. Neurol. *264*, 2345–2347.
67. Vishnempet Shridhar, S., and Christakis, N.A. (2023). Approaching disease transmission with network science. Nat. Rev. Bioeng. *2*, 6–7. https://doi.org/10.1038/s44222-023-00139-0.
68. Cantu, V.A., Sadural, J., and Edwards, R. (2019). PRINSEQ++, a multi-threaded tool for fast and efficient quality control and preprocessing of sequencing datasets. Preprint at PeerJ. https://doi.org/10.7287/peerj.preprints.27553v1.
69. BMTagger https://ftp.ncbi.nlm.nih.gov/pub/agarwala/bmtagger/.
70. Bolger, A.M., Lohse, M., and Usadel, B. (2014). Trimmomatic: a flexible trimmer for Illumina sequence data. Bioinformatics *30*, 2114–2120.
71. Lungeanu, A., McKnight, M., Negron, R., Munar, W., Christakis, N.A., and Contractor, N.S. (2021). Using Trellis software to enhance high-quality large-scale network data collection in the field. Soc. Networks *66*, 171–184.
72. Dixon, P. (2003). VEGAN, a package of R functions for community ecology. J. Veg. Sci. *14*, 927–930.
73. Beghini, F., McIver, L.J., Blanco-Míguez, A., Dubois, L., Asnicar, F., Maharjan, S., Mailyan, A., Manghi, P., Scholz, M., Thomas, A.M., et al. (2021). Integrating taxonomic, functional, and strain-level profiling of diverse microbial communities with bioBakery 3. Elife *10*, e65088. https://doi.org/10.7554/eLife.65088.
74. Kahle, D., and Wickham, H. (2013). Ggmap: Spatial visualization with ggplot2. R J. *5*, 144.
75. Kuznetsova, A., Brockhoff, P.B., and Christensen, R.H.B. (2017). LmerTest package: Tests in linear mixed effects models. J. Stat. Softw. *82*, 1–26. https://doi.org/10.18637/jss.v082.i13.
76. Balduzzi, S., Rücker, G., and Schwarzer, G. (2019). How to perform a meta-analysis with R: a practical tutorial. Health *22*, 153–160.
77. Hansen, T.F., Bolstad, G.H., and Tsuboi, M. (2022). Analyzing disparity and rates of morphological evolution with model-based phylogenetic comparative methods. Syst. Biol. *71*, 1054–1072.
78. Mallick, H., Rahnavard, A., McIver, L.J., Ma, S., Zhang, Y., Nguyen, L.H., Tickle, T.L., Weingart, G., Ren, B., Schwager, E.H., et al. (2021).

Multivariable association discovery in population-scale meta-omics studies. PLoS Comput. Biol. *17*, e1009442.

79. Revell, L.J. (2024). phytools 2.0: an updated R ecosystem for phylogenetic comparative methods (and other things). PeerJ *12*, e16505.

80. Pasolli, E., Schiffer, L., Manghi, P., Renson, A., Obenchain, V., Truong, D.T., Beghini, F., Malik, F., Ramos, M., Dowd, J.B., et al. (2017). Accessible, curated metagenomic data through ExperimentHub. Nat. Methods *14*, 1023–1024.

81. CDC (2023). Centers for Disease Control and Prevention (Centers for Disease Control and Prevention). https://www.cdc.gov/.

82. Chemistry, G. Reference range SI reference intervals SERUM. https://www.nbme.org/sites/default/files/2020-07/Laboratory_Reference_Values.pdf.

83. Williams, N. (2014). The GAD-7 questionnaire. Occup. Med. *64*, 224.

84. Kroenke, K., Spitzer, R.L., and Williams, J.B. (2001). The PHQ-9: validity of a brief depression severity measure. J. Gen. Intern. Med. *16*, 606–613.

STAR★METHODS

KEY RESOURCES TABLE

REAGENT or RESOURCE	SOURCE	IDENTIFIER
Biological samples		
Human stool samples from Honduran cohort	This Paper	N/A
Critical commercial assays		
TissueLyzer	Qiagen, Hilden, Germany	N/A
Chemagic Stool gDNA extraction kit	Perkin Elmer, Massachusetts, USA	N/A
KAPA Hyper Library Preparation	KAPA Biosystems, Massachusetts, USA	N/A
Illumina NovaSeq 6000	Illumina, California, USA	N/A
Deposited data		
Raw human gut metagenomic sequencing data	This paper	NCBI-SRA, accession number: PRJNA999635
Software and algorithms		
Prinseq lite	Cantu et al.[68]	https://github.com/uwb-linux/prinseq
BMTagger	BMTagger[69]	https://ftp.ncbi.nlm.nih.gov/pub/agarwala/bmtagger/
Trimmomatic	Bolger[70]	https://github.com/usadellab/Trimmomati
Trellis	Lungeanu et al.[71]	https://trellis.yale.edu/
MetaPhlAn (version 4.0.0)	Blanco-Míguez et al.[9]	https://github.com/biobakery/MetaPhlAn/
StrainPhlAn (version 4.0.0)	Blanco-Míguez et al.[9]	https://github.com/biobakery/MetaPhlAn/
VEGAN (version 2.3–5)	Dixon[72]	https://CRAN.R-project.org/package=vegan
HUMAnN (version 3.0.0)	Beghini et al.[73]	https://github.com/biobakery/humann
ggmap	Kahle and Wickham[74]	https://github.com/dkahle/ggmap
lmerTest (version 3.1.0)	Kuznetsova et al.[75]	https://CRAN.R-project.org/package=lmerTest
Meta (version 4.9–9)	Balduzzi et al.[76]	https://CRAN.R-project.org/package=meta
Evolvability (version 2.0.0)	Hansen et al.[77]	https://CRAN.R-project.org/package=evolvability
MaAsLin2 (version 1.0.0)	Mallick et al.[78]	https://github.com/biobakery/Maaslin2
Phytools (version 1.9–23)	Revell[79]	https://github.com/liamrevell/phytools
Available code for this study	This paper	https://github.com/human-nature-lab/Phenotype-paper. (Zenodo: https://doi.org/10.5281/zenodo.11476406)

RESOURCE AVAILABILITY

Lead contact

Further information and requests should be directed to Dr. Nicholas A. Christakis (nicholas.christakis@yale.edu).

Materials availability

This study did not generate new unique reagents.

Data and code availability

- Metagenomic sequences for the study participants are deposited in NBCI SRA and available under accession number PRJNA999635.
- The code for replicating the analysis is available at https://github.com/human-nature-lab/Phenotype-paper. (Zenodo https://doi.org/10.5281/zenodo.11476406).
- Any additional information required to reanalyze the data reported in this work paper is available from the lead contact upon request.

EXPERIMENT MODEL AND STUDY PARTICIPATION DETAILS

Sample collection, library preparation, and sequencing

Participants were instructed on how to self-collect the fecal samples using a training module and promptly returned samples to a local team which then stored them in liquid nitrogen at the collection site and then moved them to a −80 C° freezer in Copan Ruinas, Honduras. Samples were then shipped on dry ice to the United States and stored in −80 C° freezers.

Stool material was homogenized using TissueLyzer from Qiagen and the resulting lysate was prepared for extraction with the Chemagic Stool gDNA extraction kit (PerkinElmer) and extracted on the Chemagic 360 Instrument (PerkinElmer) following the manufacturer's protocol. Sequencing libraries were prepared using the KAPA Hyper Library Preparation kit (KAPA Biosystems). Shotgun metagenomic sequencing was carried out on Illumina NovaSeq 6000. Samples not reaching the desired sequencing depth of 50Gbp were re-sequenced on a separate run.

Raw metagenomic reads were deduplicated using prinseq lite (version 0.20.2[68]) with default parameters. The resulting reads were screened for human contamination (hg19) with BMTagger[69] and then quality filtered with trimmomatic[70] (version 0.36, parameters "ILLUMINACLIP:nextera_truseq_adapters.fasta:2:30:10:8:true SLIDINGWINDOW:4:15 LEADING:3 TRAILING:3 MINLEN:50").

This resulted in a total of 1,871 samples with an average read depth is 82,082,675 (SD = 812,462.4) (Figure S1). The adult population in our 19 villages ranges from 66 to 432 individuals. The average age of participants was 41 (SD = 17; range: 15–93); 63.7% were women; and 41.8% were married. The average household wealth index score (ranging from least wealthy (1) to most wealthy (5)) is 3.26 (standard deviation 1.33), measured from various household items.

Local involvement in the research

In keeping with proper standards for such research, we worked closely with the local population of Copan, sought feedback and approval from officials at the Ministry of Health (MOH) of Honduras, and endeavored to provide practical benefits to the local community. Here, we briefly summarize this history and outline some of our principles and actions in this regard.[8]

When we began designing this cohort project in 2013 (for the whole cohort of 176 villages and 24,702 people in the parent RCT), the Bill and Melinda Gates Foundation (BMGF) introduced us to the Inter-American Development Bank (IDB), which has been supporting and doing work throughout Latin America, and IDB in turn introduced us to the Honduras MOH. Because of this pathway to getting the project launched, we worked with local and regional public health organizations and with local leaders. From the outset when the original underlying cohort for this study was impaneled, we sought extensive local involvement, beginning with a needs assessment where local village residents told us about topics of concern to them in a series of meetings in villages throughout the Copan region.

We periodically briefed both the communities and the MOH about our findings. We also provided other material benefits to the local community. When we tested people for parasites as part of our study, we gave them the results of their tests and arranged for them to be treated. When we tested people for vision, we provided corrective glasses. We solicited ideas from the local community about what infrastructure improvements we could make, and we repaired many local playgrounds and clinics as a result. We arranged for an American company to provide free portable handheld ultrasound devices to the local health clinics, which was much appreciated by local providers. In terms of capacity building, we hired and trained over 100 local people and built capacity in the region.

Throughout our work in Honduras, and given the extent of local involvement at the regional and MOH levels, we endeavored to act with integrity, curiosity, and respect in all relationships.

Finally, we note that this research would not have been prohibited in the USA. This work is not likely to result in stigmatization, incrimination, or discrimination for the participants, and we have carefully safeguarded all data from threats to the privacy or security of our participants, which has constrained the individual-level data released here.

METHOD DETAILS

Taxonomic profiling and diversity analysis

Quantification of organisms' relative abundance was performed using MetaPhlAn 4,[9] which internally mapped the metagenomes against a database of ∼5.1M marker genes describing more than 27k∼ species-level genome bins (SGB).

We identified a total of 2,508 species in our dataset. Among the 2,508 species, 639 species were used for association analysis after filtering for minimum relative abundance values (10^{-2}), and a minimum of 10% prevalence in the population (n = 187).

We performed strain-level profiling for these species with StrainPhlAn 4[9](parameters: "-phylophlan_mode accurate")

Microbiome species richness was estimated using the Shannon entropy index and the total number of observed species (i.e., those with relative abundance simply greater than zero). Multidimensional scaling analysis (cmdscale R function) was performed on the Bray-Curtis dissimilarity index (vegdist function from the vegan R package[72]) calculated on the relative abundances obtained by MetaPhlAn4.

Functional potential analysis was performed using HUMAnN 3.0.[73] Gene family profiles were normalized using relative abundances and collapsed into MetaCyc pathways.

To understand the amount of variance explained by various factors, we performed a PERMANOVA analysis (adonis function from the vegan package[72]) using the "bray" method; the diversity matrix was calculated on both species-level relative abundances and MetaCYC pathway relative abundances as input, including the 123 phenotypes variables into the model. All the comparisons were run with 999 permutations.

Factor characterization

We measured a broad range of phenotypes and factors using standard measures.[7] Description and statistics on all factors can be found in Tables S2–S4. Physiological measurements were deemed within normal limits in accordance with CDC[81] and NBME[82] guidelines (Figure S3).

We used self-reported information to discern whether people were healthy or were diagnosed with various conditions. General anxiety disorder is derived from a set of 7 questions from a self-reported survey-based questionnaire though our TRELLIS software,[71] which assigns a score of 0 to "Not at all", 1 to "Several days", 2 to "More than half the days", and 3 to "Nearly every day". The scores are added up (maximum of 21) and partitioned as: Minimal or none ($\leq$5), Mild (6–10), Moderate (11–15), and Severe ($\geq$16).[83] The PHQ9 (Patient Health Questionnaire) score measuring depression was computed similarly, with the levels being: Minimal or none ($\leq$5), Mild (6–10), Moderate (11–15), Moderately severe (16–19), and Severe ($\geq$20).[84] Personality traits like Openness or Nervousness were also based on self-reported questions, where the participants were asked to rate themselves between strongly disagree to strongly agree for each of the personality questions.

The Frequency of intake of various food items was self-reported, ranging from: "Never/rarely" to "Every day". These frequencies were used as input in the diet-microbiome association model. The diet diversity score (DDS)[19] was calculated by classifying individual food types into one of the following categories: cereals, roots/tubers, vegetables, fruits, meat/poultry/offal, eggs, fish/seafood, pulses/legumes/nuts, milk and dairy products, oils/fats, or sugar/honey. If any of these food items were consumed daily, the respective categories would get 1 for that individual. The sum across these categories would define the DDS score of this individual. The maximum possible DDS score would be 11 and the minimum would be 0.

Numerical values were reported for alcohol frequency and cigarette frequency. The daily alcohol intake ranged from "1 or 2" to "10 or more" drinks. Cigarette usage was reported as a "Yes" or "No".

The household wealth index is computed using Multiple Correspondence Analysis (MCA) based on all the household items. The index ranged from 1, indicating low wealth, to 5, indicating high wealth.

We explored associations with several social network features, including the degree, transitivity, and betweenness centrality of each individual. To uncouple the effects of kin and non-kin social connections, we investigated microbiome associations in familial networks, friendship networks, and combined networks. In the combined network, we computed the amount of kin in a person's first 3 degrees of social connections (i.e., among a person's friends, friends of friends, and friends of friends of friends) to assess the relative effect of having kin close to a person within the social network. In addition to kin and non-kin relationships, we also explored the microbiome's association with cohabiting partners.

Population-weighted village centroid

We collected the GPS coordinates (latitude and longitudes) of all the building in the village. Since multiple individuals can reside in a building, the population-weighted centroid was chosen as the reference center of the village, which was then used to compute every individual's distance from this village center. Satellite plots were created using "ggmap" package in R.[74]

QUANTIFICATION AND STATISTICAL ANALYSIS

Model for microbiome-factor regression

For the association model with species-level microbiome and the factors, a linear mixed-effects model was used to explore the relationship of the variability in the factors and the variability in the microbiome. The linear mixed-effect models were created using the lmerTest R package (v 3.1).[75]

For every species and phenotype pair, we computed the following model:

$$\text{Species abundance} \sim \text{Factor of interest} + \text{Age} + \text{Sex} + \text{BMI} + \text{Batch Effect} + \text{Bristol Stool Scale} \\ + \text{DNA concentration} + \text{Sampling date} + (1 \mid \text{Village ID})$$

Species-level relative abundances were transformed using the CLR (Centered-Log Ratio) and used as input.

Since basic demographic attributes (age, sex), technical factors (DNA concentration, sequencing batch, sampling date), and BMI and Bristol stool scale accounted for most of the species and pathway variation, we used those variables as primary controls in our association models (Figures S5 and S9).

Furthermore, all associations were corrected for both microbiome species and factor using multiple hypothesis testing (Benjamini-Hochberg correction) and all significant associations are corrected for a FDR (False Discovery Rate) < 0.05.

Meta-analysis of BMI across non-Western cohorts

We screened publicly available datasets using the curatedMetagenomicData package (v3.6.2)[80] to look for cohorts from similar populations and sharing the most number of available metadata. We identified a total of 5 non-western studies having in common BMI[6,29,30,34,35] along with 4 western cohorts[28,31–33] amounting to 5,001 samples. Data was downloaded from NCBI SRA using the accessions available through 'curatedMetagenomicData' and processed using the same pipeline described before.

We then performed a meta-analysis on BMI values using species-level relative abundances using. Age, gender, and lifestyle category were used as controls. We discretized age by binning the value into three levels: child-adolescent (<18), adult (18–60), and senior (>60).

Also, a random effect meta-analysis was performed using species-level relative abundances normalized with CLR using the meta package (v 4.9–9,[76]). After using linear model to obtain correlation coefficients, the metacor function (from meta package) was used to Random effects using Paule-Mandel estimator method. *p*-values obtained were adjusted using FDR (Benjamini-Hochberg corrected). In total, 21 species were found significant after corrections. The full results are available in Table S11.

Strain-factor analysis and phylogenetic signal

For strain-level analysis, we used the Almer function from the "evolvability" R package (v 2.0.0).[77] Almer incorporates phylogenetic trees in mixed linear models as a correlated random effects structure.

$$\text{Species abundance} \sim \text{Phenotype of interest} + \text{Age} + \text{Sex} + \text{BMI} + \text{Batch effect} + \text{Bristol Stool Scale} \\ + \text{DNA concentration} + \text{Sampling date} + (1 \mid \text{Village ID}) + (1 \mid \text{phyl})$$

where, "phyl" is the variance-covariance matrix calculated from the species' phylogenetic tree. To evaluate the strain-phylogenetic effect, we compared beta coefficients from this model and the same model without the random effect on the variance-covariance matrix.

The phylogenetic signal was estimated using the "phylosig" function in "phytools" R package (v 1.9–23)[79] using the 'lambda' method. Overall, among the 78,597 species-phenotype pairs (639 species and 123 phenotypes), 52,864 pairs were chosen after filtering for phylogenetic signal. The phylogenetic signal was estimated for the phylogenetic tree of each species versus the phenotype of interest.

Polymorphic sites analysis

For polymorphic sites, files suffixed with ".polymorphic" in StrainPhlAn 4 output were used after discarding 0's in the "percentage of polymorphic sites" column so as to discard subjects without the species of interest. Wilcoxon rank-sum tests were performed across categories within phenotypes to check for significant changes in polymorphic sites. In addition, linear regression was also performed to investigate the relationship between polymorphic site percentage and individual host phenotypes (see Table S10).

Differential abundance analysis

We used MaAsLin2 (v 1.0.0)[78] to determine the association between species and disease status (healthy or unhealthy) of individuals and to estimate the effect sizes and *p*-values. Statistically significant species were retained. Species-level relative abundances were normalized using CLR and used as input for MaAsLin2. Age, sex, BMI, DNA concentration, sampling date, and Bristol stool scale were used as fixed-effect controls and village as a random effect control. All the resulting *p*-values obtained from the MaAsLin2 models were corrected for multiple hypothesis testing using FDR.

Cell Reports, Volume *43*

Supplemental information

Environmental, socioeconomic, and health factors associated with gut microbiome species and strains in isolated Honduras villages

Shivkumar Vishnempet Shridhar, Francesco Beghini, Marcus Alexander, Adarsh Singh, Rigoberto Matute Juárez, Ilana L. Brito, and Nicholas A. Christakis

Figures List:

1. **Honduran Gut microbiome description**
2. **Phenotype-phenotype correlation.**
3. **Phenotype-microbiome association clustering.**
4. **Relationship between health and microbiome.**
5. **Variance explained.**
6. **Alpha diversity of individuals exposed to animals.**
7. **Diet diversity score.**
8. **Comparison of species and strain models.**
9. **Principal Coordinates Analysis (PCoA) of the full Honduran cohort.**

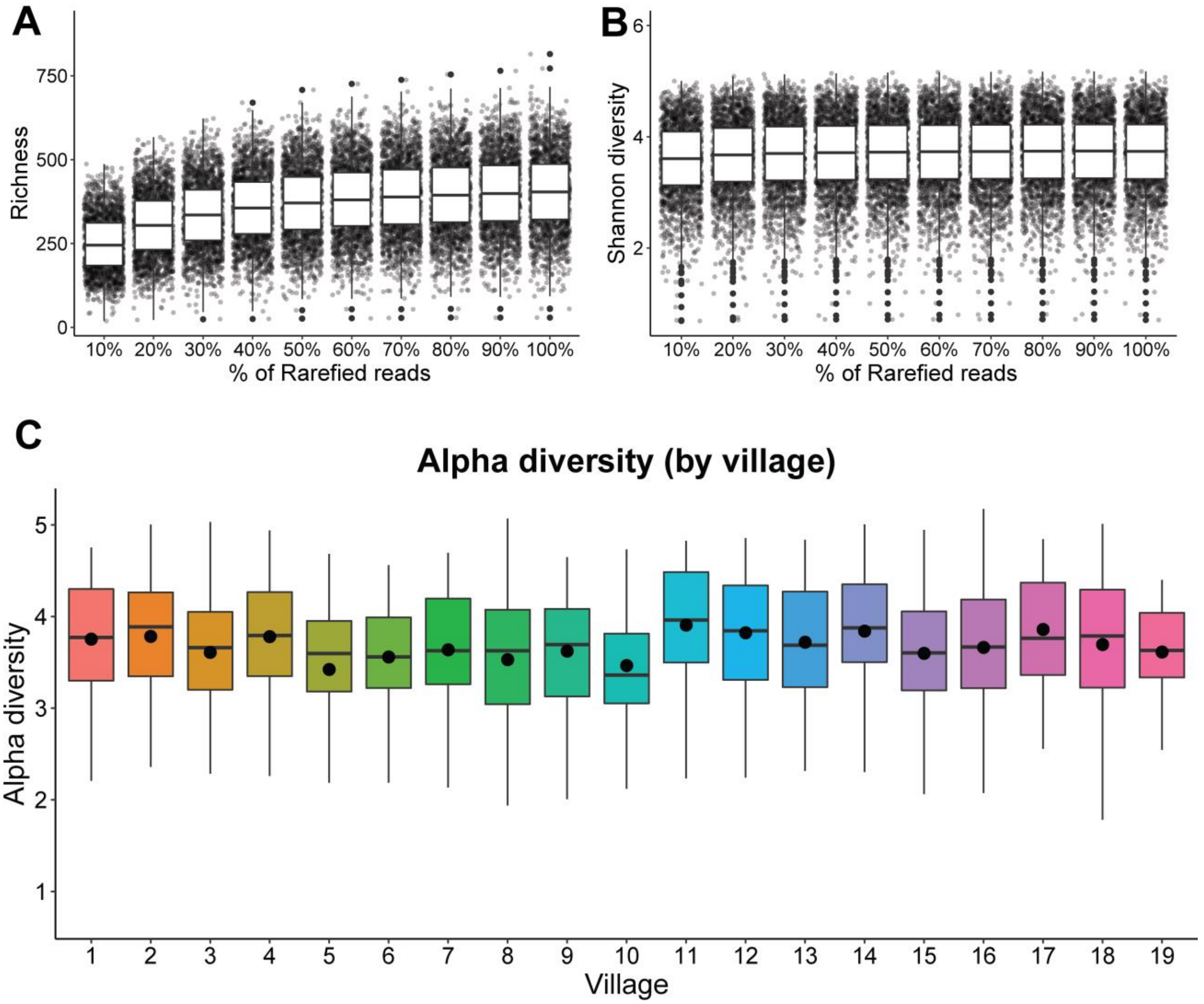

Figure S1 Honduran Gut microbiome description (Related to STAR methods). (A) Collector's curve of species richness (number of species per sample) across varying percentages of rarefied reads for all 1,871 Honduran samples (in light dots and box plot) show an increasing trend followed by a plateau at 60% of the all the rarefied reads. The average species richness is around 380 (plateau). (B) Collector's curve of alpha-diversity vs percentage of read-depth across all 1,871 Honduran samples show a flat trend. (C) The overall average of Alpha diversity across cohort is 3.7 and there is no village statistically different in distribution of alpha diversity compared to any other village (Wilcoxon rank-sum test). Black dots indicate mean alpha diversity for each village.

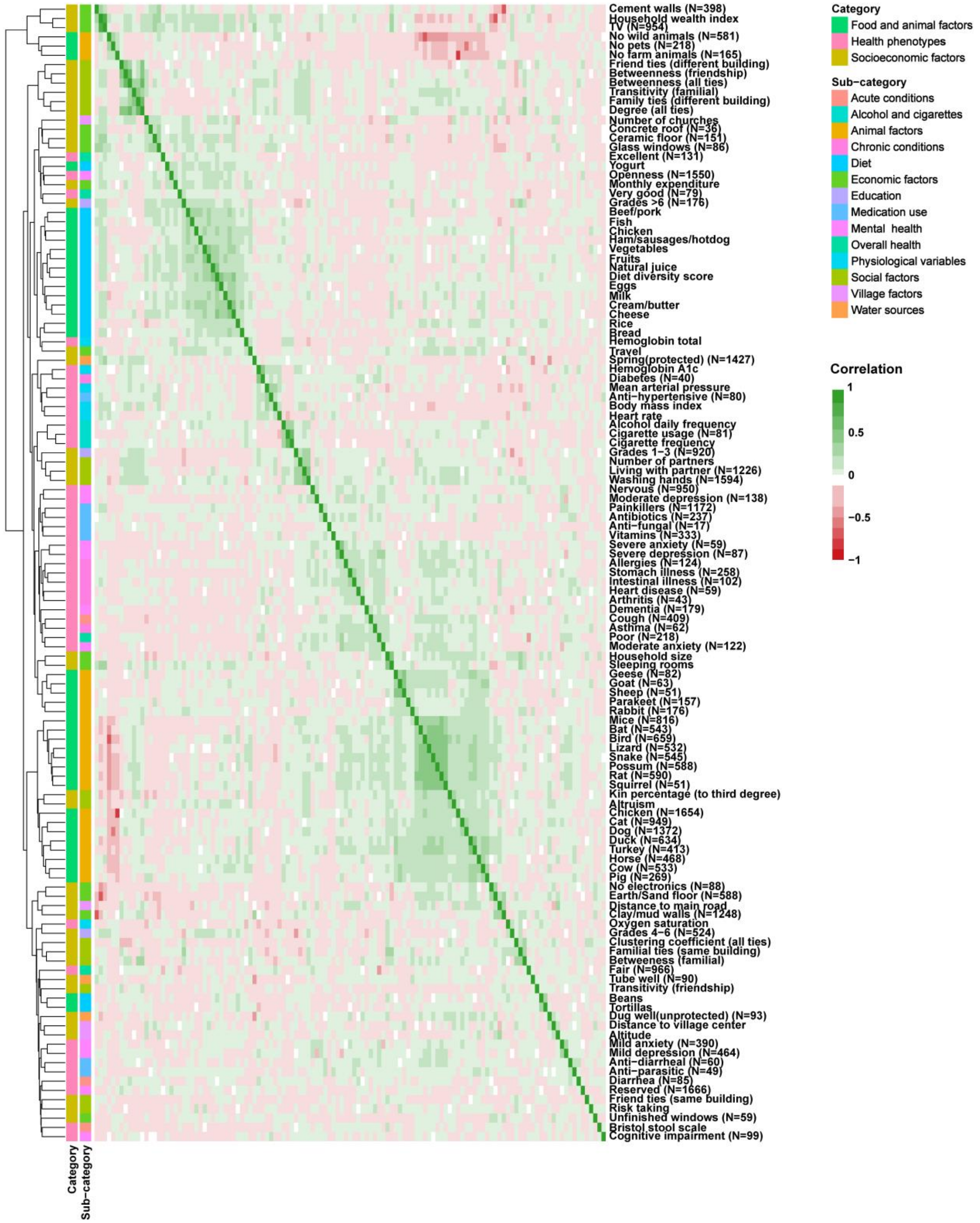

Figure S2 Phenotype-phenotype correlation (Related to Figure 2). (A) A matrix showing raw correlations between the phenotypes from every category (health, food and animals, socioeconomic factors). Column names are the same as the row names indicated on the right side of the matrix. Color ranges from positive (green) to negative (red) correlations. The correlations are also clustered according to the hierarchical clustering and annotated according to broader category or sub-category of phenotypes and factors (see **Supplementary table 1**).

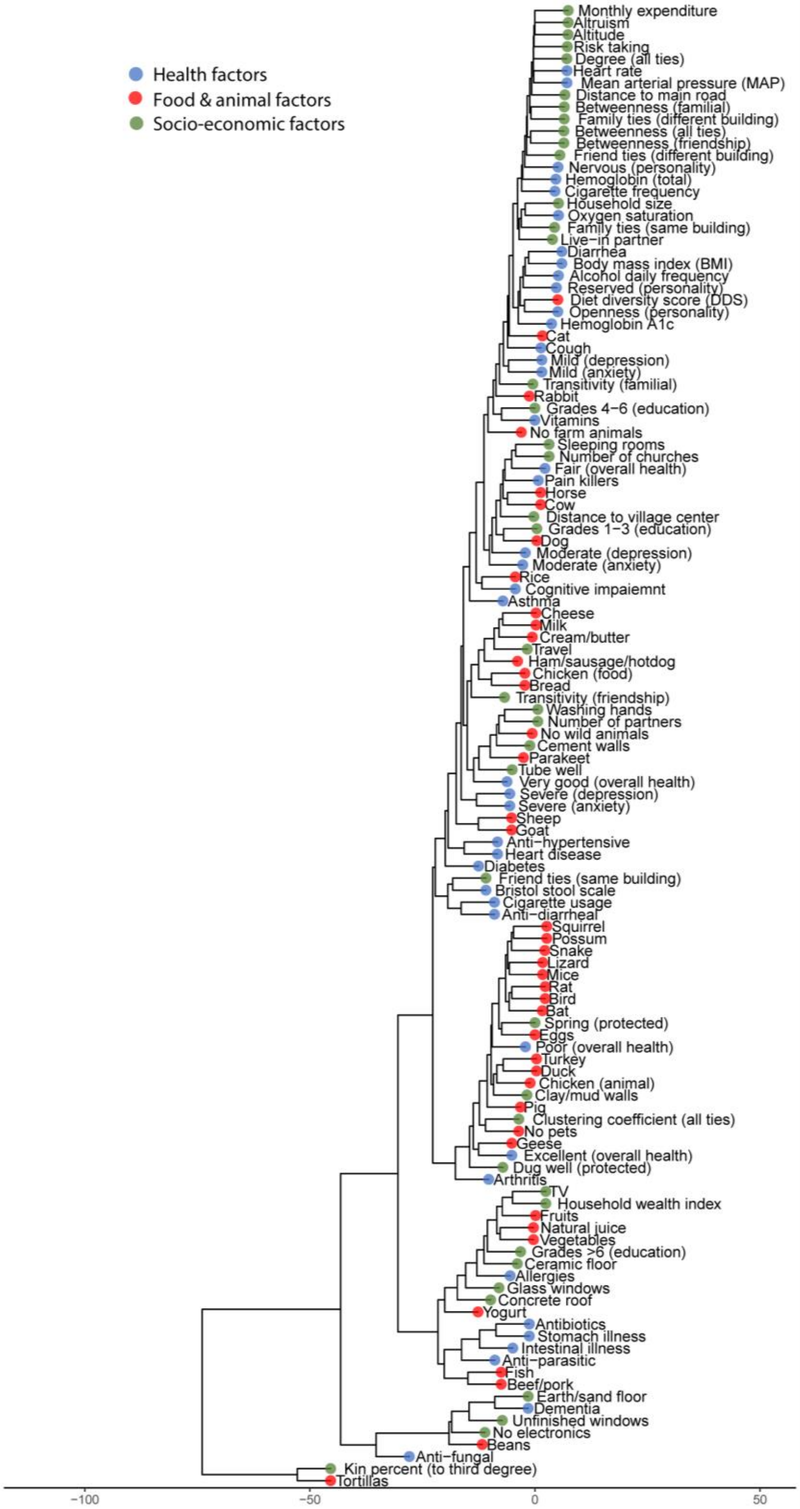

Figure S3 Phenotype-microbiome association clustering (Related to Figure 2). Effect sizes from associations of all 123 phenotypes with 639 species are hierarchically clustered with respect to phenotypes. This phenotype tree is another representation of how similarly behaving a pair of phenotypes are with respect to how they associate with the gut microbiome overall.

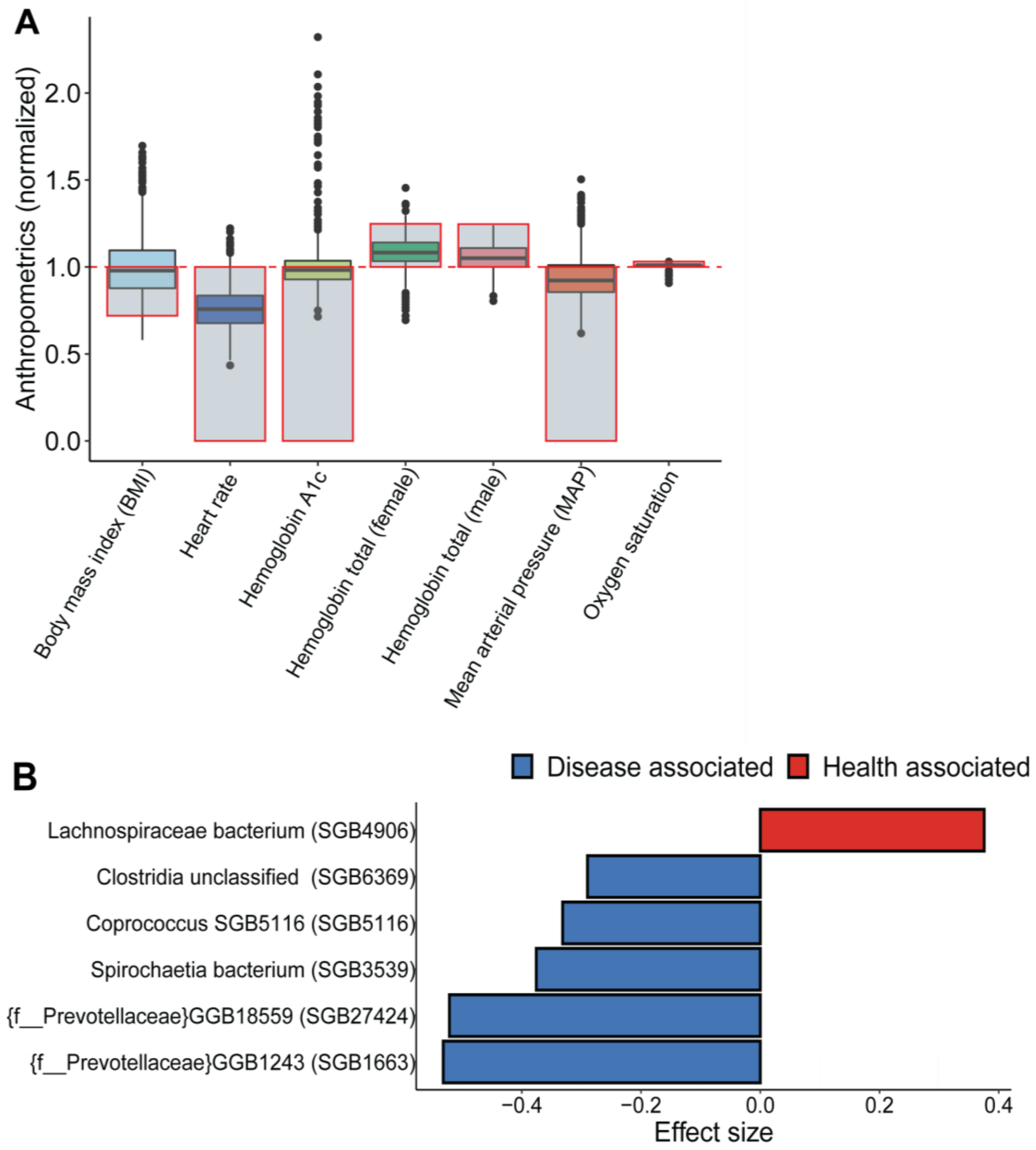

Figure S4 Relationship between health and microbiome (Related to STAR methods). (A) Graphical visualization of physiological measurements (anthropometrics) of all N=1,871 villagers, with the grey box indicating normal values of each respective physiological measurement. The red box indicates the bounding limit of healthy ranges. (B) In the entire cohort, there were 468 chronically diseased individuals (who had at least one chronic condition). Differential abundance in healthy vs chronically diseased individuals using MaAsLin2 (see **Methods**) shows six significant species (after FDR correction of p-values). One of them (*Lacnospiraceae bacterium*) was differentially abundant in healthy individuals. On the other hand, five species (uSGB1663 and uSGB27424 from the *Prevotellaceae* family, *Spirochaetia bacterium*, *Coprococcus*, and uSGB6369 from the *Clostridia* family), two of which are unknown, were differentially abundant in chronically diseased individuals.

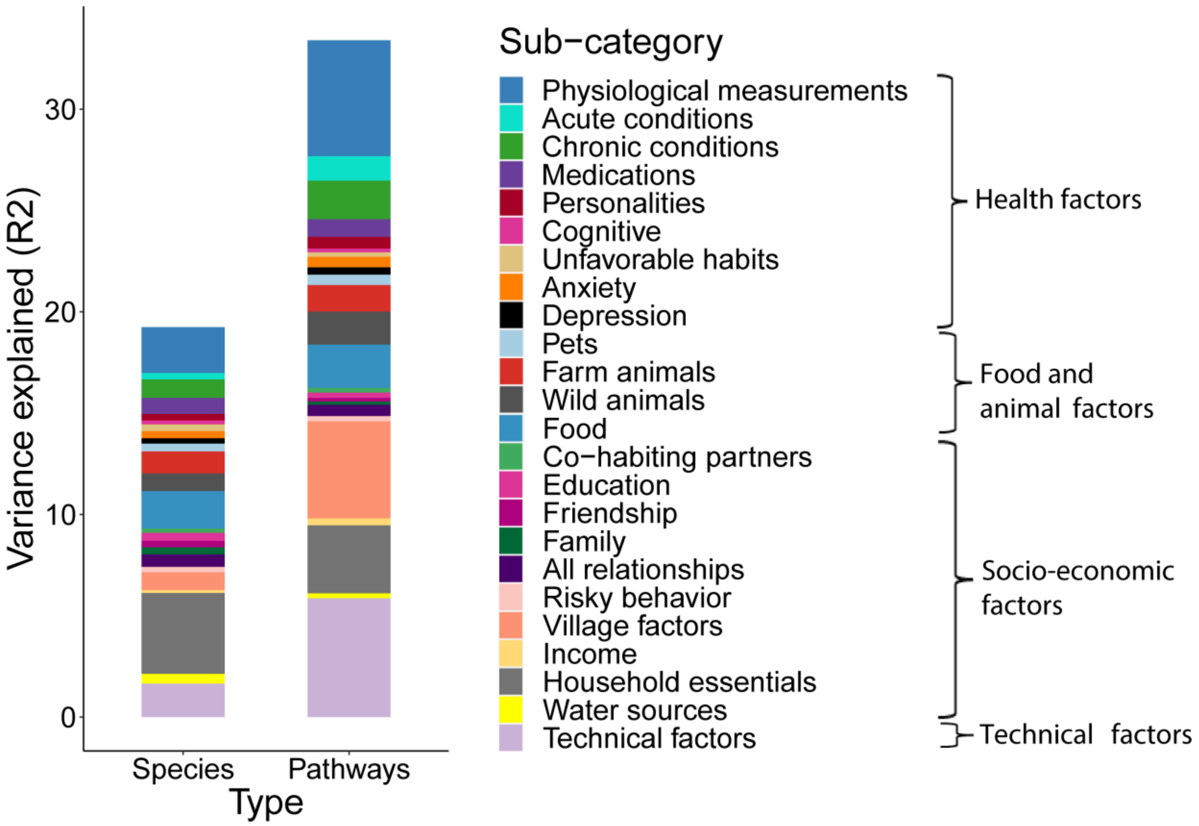

Figure S5 Variance explained (Related to Figure 2). PERMANOVA analysis (999 permutations, p-value<0.001) computed on all phenotypes shows the variance explained in species and pathway compositions with a breakdown of sub-categories of all phenotypes (health, food and animal, socioeconomic factors). Overall, all the phenotypes together explain 19.2% and 33.4% of the variance explained in species and pathways, respectively. "Technical factors" here include age, sex, DNA concentration, sequencing batch, and sampling date. (See **Supplementary Table 7** for complete breakdown of variance explained in each sub-categories)

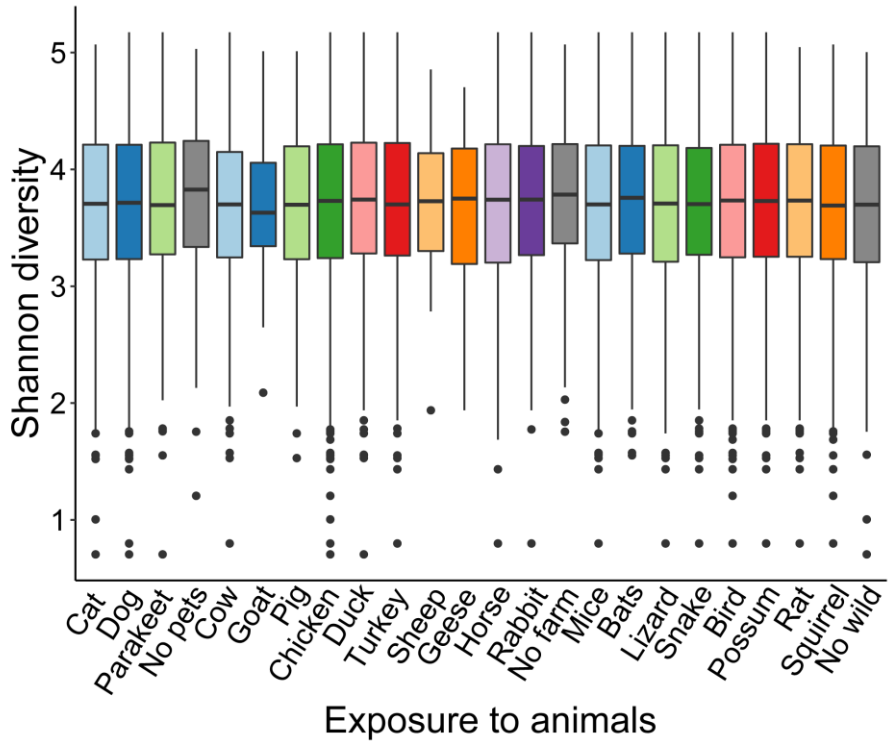

Figure S6: Alpha diversity of individuals exposed to animals (Related to Figure 2). Shannon diversity distribution among villagers exposed to pets, farm animals, and wild animals shows no significant differences between groups.

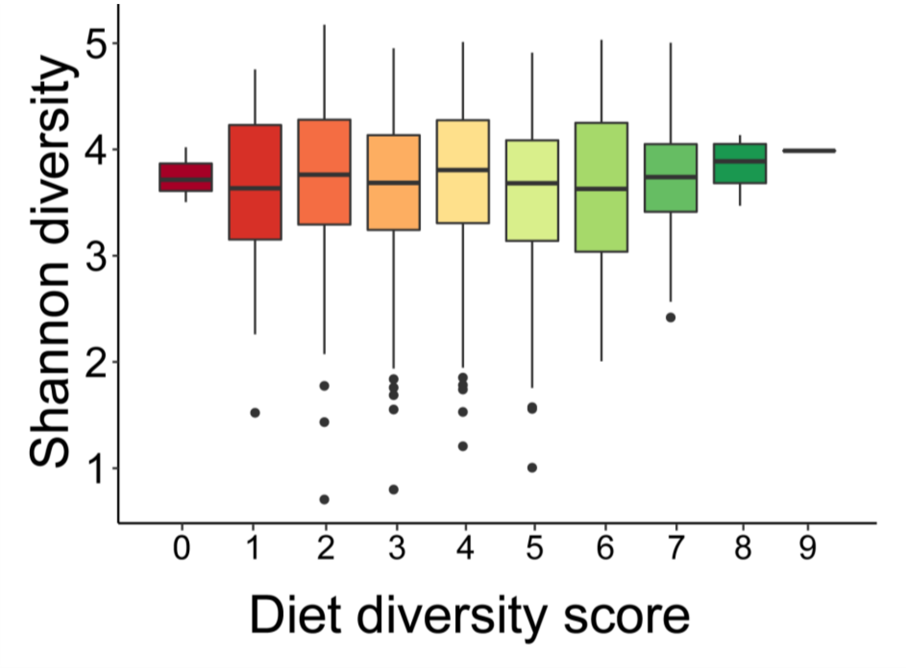

Figure S7: Diet diversity score (Related to Figure 2 and STAR methods). Boxplot showing the Shannon diversities of individuals with varying diet diversity scores (see **STAR Methods** for calculation of DDS scores).

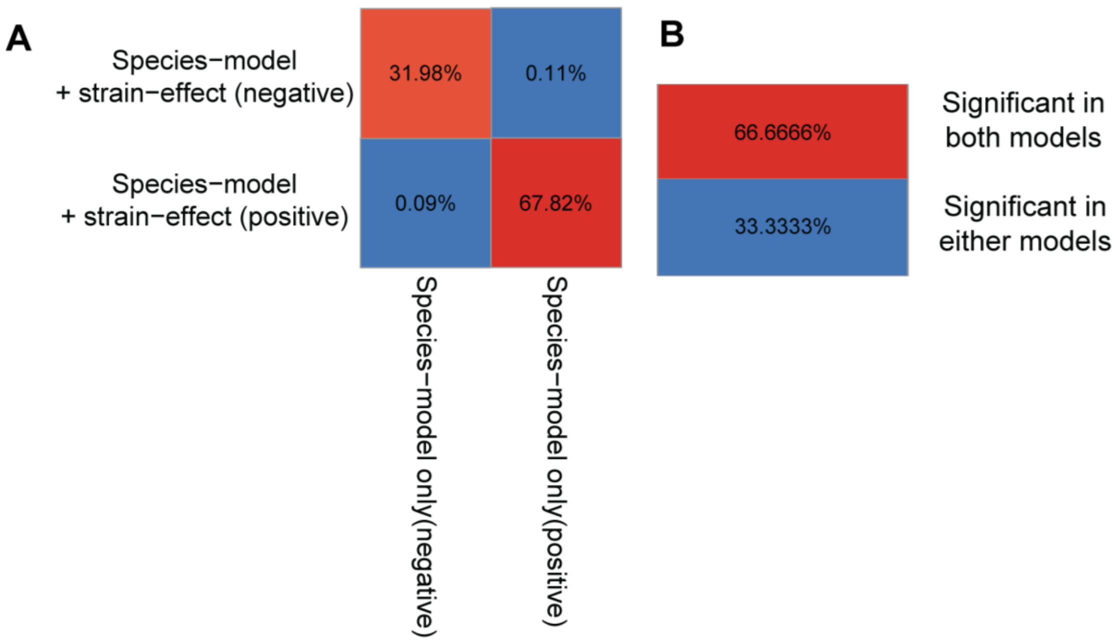

Figure S8: Comparison of species and strain models (Related to Figure 4). (A) Side-by-side comparison of the direction of associations in both models (with and without strain-phylogenies). Each quadrant indicates positive or negative associations in either model. (B) Figure showing the presence of significant associations in both models compared to their presence in either of the models.

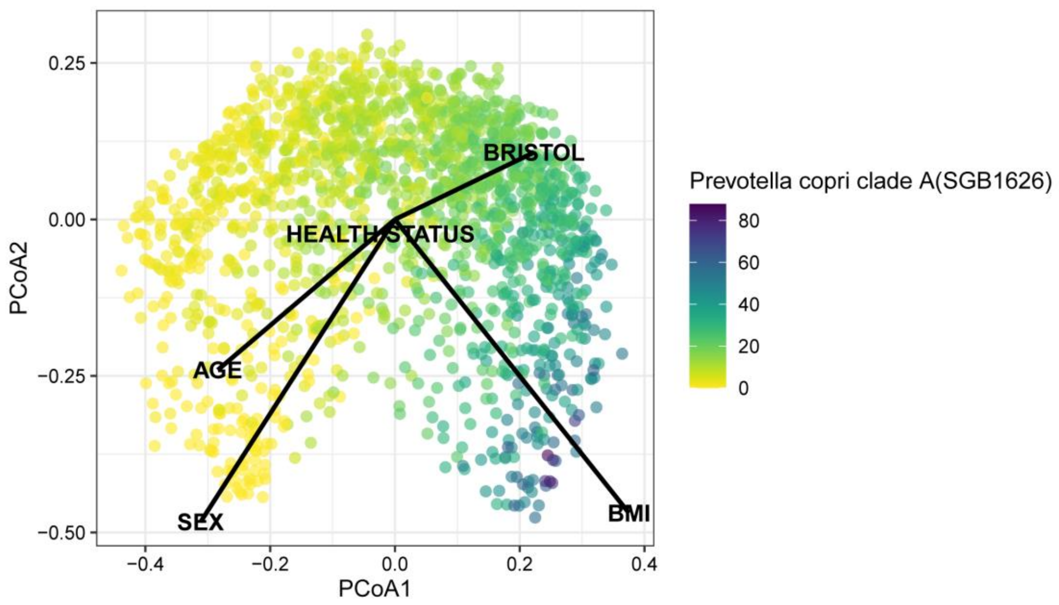

Figure S9: Principal Coordinates Analysis (PCoA) (Related to STAR methods). PCoA plot of the overall gut microbiome computed across 1,871 samples using the species-level relative abundances (legend) generated by MetaPhlAn4. Health status, age, sex, body mass index (BMI), and Bristol stool scale are shown as arrows along with the direction of influence. Samples are colored with the relative abundances of *Prevotella copri* (clade A).
